# Quantifying inherent predictability and spatial synchrony in the aphid vector *Myzus persicae*: field‐scale patterns of abundance and regional forecasting error in the UK

**DOI:** 10.1002/ps.7292

**Published:** 2022-12-19

**Authors:** James R. Bell, Suzanne J. Clark, Mark Stevens, Andrew Mead

**Affiliations:** ^1^ Rothamsted Insect Survey Rothamsted Research West Common Harpenden UK; ^2^ Statistics and Data Science Rothamsted Research Harpenden UK; ^3^ BBRO, Centrum Norwich Research Park Norwich UK

**Keywords:** *Beta vulgaris*, sugar beet, virus reservoirs, yellow water traps, spatial synchrony, weighted permutation entropy

## Abstract

**Background:**

Sugar beet is threatened by virus yellows, a disease complex vectored by aphids that reduces sugar content. We present an analysis of *Myzus persicae* population dynamics with and without neonicotinoid seed treatment. We use 6 years' yellow water trap and field‐collected aphid data and two decades of 12.2 m suction‐trap aphid migration data. We investigate both spatial synchrony and forecasting error to understand the structure and spatial scale of field counts and why forecasting aphid migrants lacks accuracy. Our aim is to derive statistical parameters to inform regionwide pest management strategies.

**Results:**

Spatial synchrony, indicating the coincident change in counts across the region over time, is rarely present and is best described as stochastic. Uniquely, early season field populations in 2019 did show spatial synchrony to 90 km compared to the overall average weekly correlation length of 23 km. However, 70% of the time series were spatially heterogenous, indicating patchy between‐field dynamics. Field counts lacked the same seasonal trend and did not peak in the same week. Forecasts tended to under‐predict mid‐season log_10_ counts. A strongly negative correlation between forecasting error and the proportion of zeros was shown.

**Conclusion:**

Field populations are unpredictable and stochastic, regardless of neonicotinoid seed treatment. This outcome presents a problem for decision‐support that cannot usefully provide a single regionwide solution. Weighted permutation entropy inferred that *M. persicae* 12.2 m suction‐trap time series had moderate to low intrinsic predictability. Early warning using a migration model tended to predict counts at lower levels than observed. © 2022 The Authors. *Pest Management Science* published by John Wiley & Sons Ltd on behalf of Society of Chemical Industry.

## Graphical abstract

Migrating populations of the aphid vector, *Myzus persciae*, have moderate to low intrinsic predictability. Field populations are stochastic, regardless of neonicotinoid seed treatment presenting a problem for decision‐support.

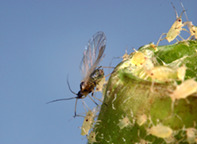

## 1 INTRODUCTION

Sugar beet (*Beta vulgaris*) accounts for 20% of the world's sugar production.^1^ In England, 100 000 ha of arable land produces 8 million tons of beet annually, meeting more than half of domestic sugar demand.^2^ However, in both the UK and across the EU, yields are threatened by virus yellows (VY), a disease of sugar beet that comprises a complex of *Polerovirus* and *Closterovirus* types that decrease the ability of the infected leaf to photosynthesize, therefore reducing sugar yield.^3^, ^4^ VY infection begins when aphids vector the virus through the leaf tissues following seedling emergence. Three weeks after transmission, symptoms are expressed and yellowing of the leaves then follows.^5^

Beet mild yellowing virus (BMYV) and beet chlorosis virus (BChV) are persistent and belong to the *Polerovirus* genus. These viruses are vectored by two aphid species, *Myzus persicae* (Sulzer) and, to a lesser extent, *Macrosiphum euphorbiae* (Thomas).^3^, ^4^ Although these two aphids remain infected with these viruses for their whole lifespan, beet yellows virus (BYV), a semi‐persistent *Closterovirus*, is more damaging to UK agriculture even though it only persists within the aphid host for a matter of days.^3^, ^4^ Again, the main vector is *M*. *persicae*, but the aphid *Aphis fabae* (Scopoli) can also contribute to BYV transmission, acquiring the virus within a minimum of 5 minutes of feeding on an infected host, although the probability of successful transmission increases with time spent feeding.^6^, ^7^

VY impact on sugar beet production has been intensely studied since the 1950s, but disease vector control was limited until the 1990s. Control of the main vector *M. persicae* by systemic use of neonicotinoid seed treatments from the mid‐1990s dominated agricultural strategy until 2018, when the EU implemented a change in regulations, effectively banning the active substances imidacloprid, clothianidin and thiamethoxam.^5^, ^8^ The change in policy was driven by the negative impacts of these seed treatments on pollinators, including sublethal changes in behavior of honeybees and bumble bees.^9^, ^10^ Growers experienced average yield losses of 38% in the absence of the seed treatment in 2020, a loss which was valued at £43 million rising to 100% losses in parts of Cambridgeshire.^11^, ^12^

The ability to colonize numerous hosts without specialization is key to understanding the threat posed by *M. persicae*, a highly polyphagous aphid with over 40 plant families (>100 sp) within its host range, including important crop families *Amaranthaceae*, *Asteraceae*, *Brassicaceae* and *Solanaceae*.^13^, ^14^ Most agricultural “weeds” that are reservoirs of the virus (*e.g. Capsella bursa‐pastoris*) are widely distributed at the 1 km‐grid scale and may act as effective “green bridges” into sugar beet, although a minority are otherwise patchy and may be less important (*e.g. Spergula arvensis*).^15^, ^16^

Since the 1960s, various decision support tools predicting the impact of VY have been deployed to help sugar beet growers reduce prophylactic use of insecticides.^17^, ^18^ Harrington *et al*.^17^ was the first to produce a statistical forecast using aphid migration data from Rothamsted Insect Survey (RIS) 12.2 m suction‐traps. The migration model responses were first flight and log total count to the 17th June, which were shown to be driven by January–February mean temperatures. Harrington *et al*.^17^ showed an association between the VY risk to sugar beet and migration activity, with an elevated risk associated with earlier flights and the associated higher  log total counts.

Although the aphid migration component relates to the VY incidence at a regional level^18^ and migrating aphids across the 12.2 m suction‐trap network are highly synchronized,^19^, ^20^, ^21^, ^22^ this does not necessarily imply that the aphids at the field‐scale are behaving similarly.^23^ Hence, we investigate the spatial synchrony of the aphid counts across the east of England using yellow water traps (YWTs) and field counts, a network of traps that covers more than 100 000 ha of sugar beet and has been operational since 2014. Whilst the null hypothesis is of spatial randomness, we anticipate that neighboring fields will likely be more similar than those further apart, whereby the strength of synchrony declines as a function of distance. Our research spans a period of neonicotinoid seed treatment from 2014 to 2018 and then a shorter 2‐year period without seed treatment. We test the hypothesis that spatial synchrony declines with distance. We also use generalized additive mixed models (GAMMs) to support the spatial synchrony analyses using the same YWT data, providing further insight into the seasonal occurrence and spatial patterning in each year, testing for the presence of spatial clines in abundance and for evidence of temporal autocorrelation, which would reveal the level of predictability of populations in time. We also conduct an analysis of the forecasting error associated with predicting the total count of migrating aphids to 17th June using linear regressions using the Brooms Barn suction‐trap that heuristically informs an approximate radius of 80 km around the trap (20 000 km^2^, 10% of the land area of the East of England), subject to wind speed, landscape and elevation change. Whilst there are strong relationships between long‐term aphid data from the RIS 12.2 m suction‐trap network and long‐term weather data that demonstrate that the timing of spring first flight migrations is predictable,^17^, ^21^ we assess the forecasting accuracy to predict numbers of aphids against those that were subsequently observed in the 12.2 m suction‐trap at Brooms Barn, Suffolk. We attempt to explain the discrepancy using model‐free permutation entropy.^24^

## 2 MATERIALS AND METHODS

The study was conducted in the UK, in the main sugar‐beet growing region that is spread across the East of England (52.623, 1.220) and further north in East Midlands (53.089, −0.817) regions.

### 2.1 Yellow water traps

YWTs are an effective tool for monitoring aerial pest activity within agricultural systems and have been used specifically to study aphid migration and threats to crops since the 1950s.^25^, ^26^

**Figure 1:**
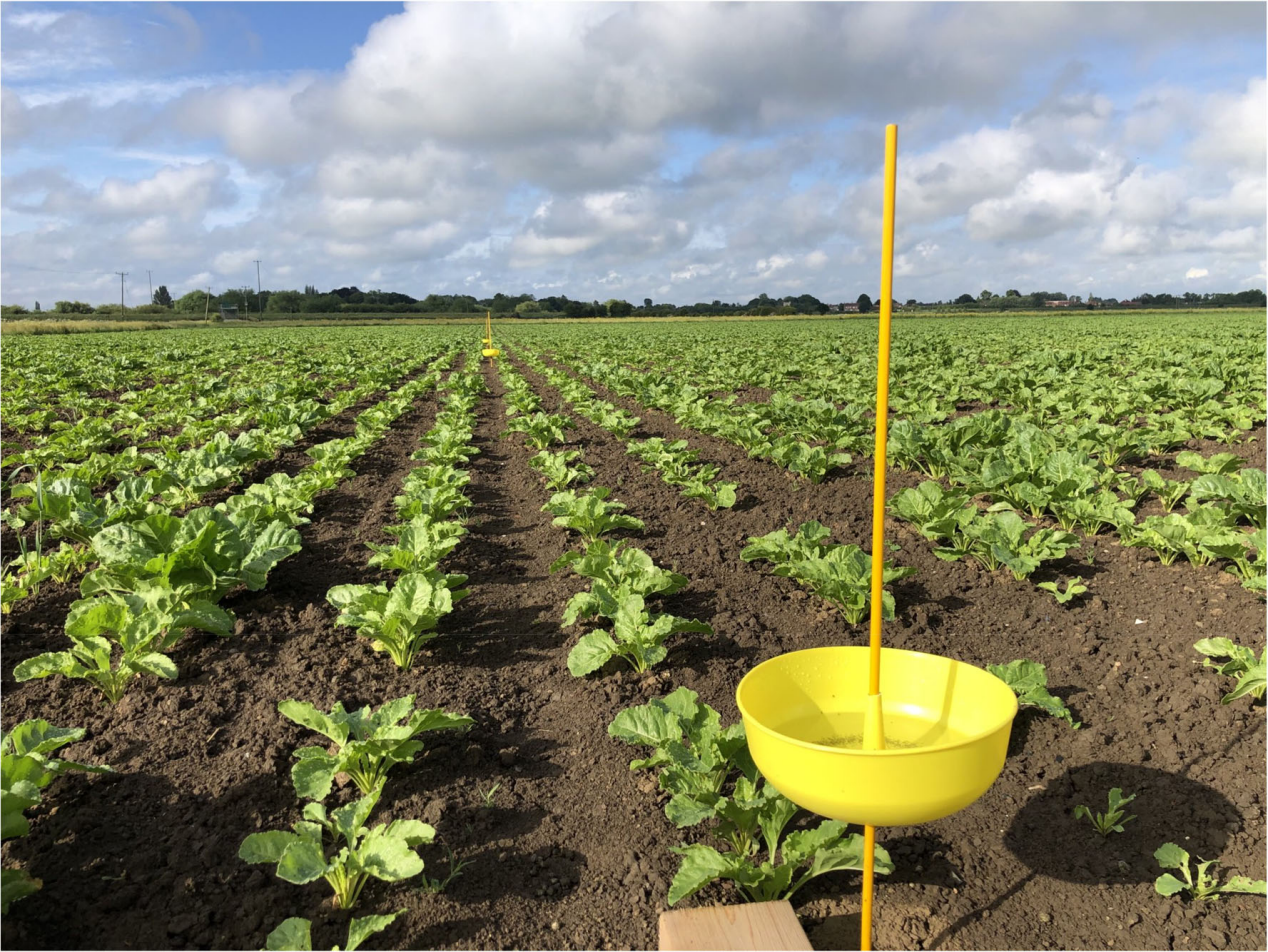
Three yellow water traps (27 cm ø, Flora Insect Trap), spaced approximately 15 m apart and at least 15 m from any field margin. Traps were continually adjusted to crop height throughout the season. Photo credit: BBRO.

In each year from 2014 to 2019, a network of YWTs was deployed, organized by BBRO and managed locally by British Sugar plc contract managers or growers and agronomists across East Anglia. In each field, three YWTs (27 cm ø, Flora Insect Trap), adjusted continually to crop height and approximately 15 m distance apart, were located at least 15 m from the headland of the sugar beet field (Fig. 1). Each trap was filled with water to which a small amount of detergent was added. YWTs began operation between mid‐April and early May, depending on year, but always before the aphid season began. Catches were collected every Monday and Thursday throughout the aphid season; on those days, the three trap catches within each field were amalgamated to represent a field count and then sent to BBRO for identification. Trapping lasted past peak aphid migration and when crops had reached full canopy expansion, at which point they likely had acquired mature plant resistance to feeding and aphids were no longer monitored.

The field counts were treated as accumulated catches. These were first apportioned *pro rata* to their component days and then the daily counts were summed by week to give a total aphid count per field‐week (week 1 = 1–7 Jan, 2 = 8–14 Jan etc.). These weeks were aligned with the 7‐day periods that have historically been used by the RIS since 1964, referred to as “standard” weeks. Using the standard week approach, the season could be systematically assessed across years and sites. Specifically, the season started particularly early in 2019, but in other years, aphids were largely absent in weeks 17–18 and were not analyzed. In 2014 and 2019, the aphid season had finished by week 27, but in all other years, aphids were recorded until week 29. Hence, the YWT season in terms of start and end weeks contributing to spatial analyses were: 2014 = 19–27; 2015–2018 = 19–29; 2019 = 17–27. Even then, the GAMMs were unable to process 2 weeks^19^, ^20^ in 2015 because these data were too sparse to produce prediction surfaces (see Tables 1 and 3).

**TABLE 1:** Summary of the multivariate and univariate spline correlograms used to generate annual and weekly YWT spatial synchrony parameters including the local covariance function (LCF) and the correlation length (CorL)

YWT	2014		2015		2016		2017		2018		2019	
Model type	LCF	CorL	LCF	CorL	LCF	CorL	LCF	CorL	LCF	CorL	LCF	CorL
Annual spatial synchrony	0.10	47.73	0.02	48.92	0.09	27.62	0.00	22.96	0.08	127.25	0.35	89.56
Weekly spatial synchrony												
Week 18											0.50	48.24
Week 19	0.00	0.00	−0.15	0.00	−0.20	0.00	−0.19	0.00			0.73	64.66
Week 20	0.00	0.00	−0.01	0.00	0.16	**59.03**	0.28	37.22			0.58	58.80
Week 21	−0.01	8.80	0.10	52.26	0.09	39.67	0.05	28.50			0.84	**71.30**
Week 22	−0.06	0.00	0.31	**56.45**	−0.11	0.00	−0.08	0.00	0.13	**48.63**	−0.14	0.00
Week 23	0.48	**60.32**	0.04	47.80	0.04	11.51	0.01	**119.22**	−0.24	0.00	0.11	13.63
Week 24	−0.05	0.00	0.12	37.17	−0.13	0.00	−0.16	0.00	−0.02	0.00	−0.09	0.00
Week 25	0.02	18.52	0.09	38.01	0.00	−0.00	0.03	20.14	−0.02	0.00	−0.04	0.00
Week 26	0.14	22.38	0.10	50.15			0.04	48.22	−0.10	0.00		
Week 27	−0.08	0.00	0.08	45.88			−0.66	0.00	−0.02	0.00		
Week 28									0.00	0.00		

*Note*: The peak in the weekly CorL is highlighted in bold and the average CorL across all annual models = 60.67 km and across weekly models = 23.50 km. Grey boxes indicate data were too sparse to run a model.

### 2.2 Crop inspections

In 2020, due to the coronavirus pandemic, YWT samples were not able to be analyzed at BBRO labs, therefore an alternative survey method was deployed. Forty‐one sites across East Anglia were managed by British Sugar contract managers and growers. At the start of the survey period, two rows of 10 plants were selected, at least 15 m from the field boundaries and at least 50 m apart. These same 20 plants were examined every Monday and Thursday from 16 April to 2 July and the number of winged and wingless aphids recorded. Twenty‐three counts were recorded in these 12 weeks, ending once the crop had reached full canopy cover and the main aphid migration had ended. The same standard weeks procedure, described above, was used for these data.

### 2.3 Suction‐traps

Suction‐traps continuously measure the aerial density of flying aphids at the logarithmic mean height of aphid flight (12.2 m), providing standardized daily records during the main aphid flying season.^21^ The Brooms Barn 12.2 m suction‐trap was used because it is a long‐running site in the center of the East Anglian sugar beet growing region and has an unbroken time series. Daily *M. persicae* counts from the core migration period between weeks 19–25 were used for each year between 2014–2021, matching the YWT/crop inspection time series. We also used a much longer time series from this trap (2002–2021) to estimate the forecasting error between observed and predicted *M. persicae* logged counts.

### 2.4 Statistical methods

#### 2.4.1 Spatial synchrony

**FIGURE 2:**
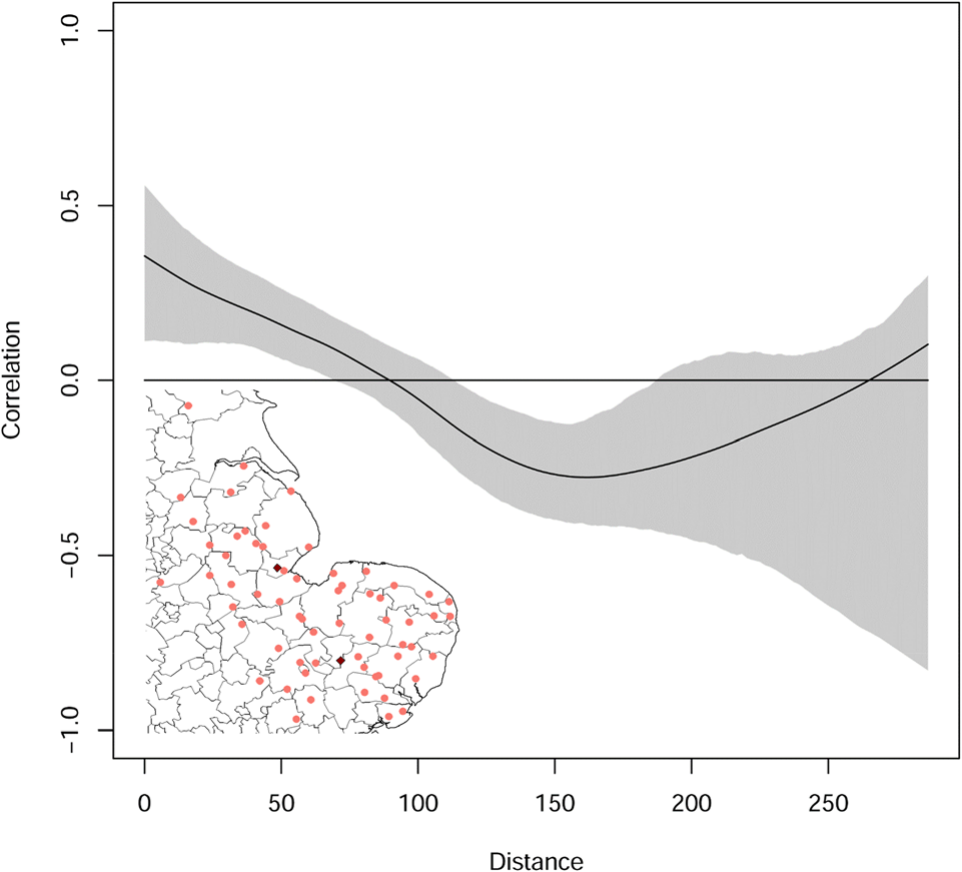
Regional measure of spatial synchrony using a multivariate spline correlogram on 2019 data using 64 sites. The local covariance function (LCF) at the intercept on the *Y*‐axis is 0.35, the correlation length (CorL) is 89.56 km on the *X‐*axis and the spatial extent is 0–290 km. Embedded is a regional site map of the YWT network used to produce the model (pink circles) and the location of suction‐traps (maroon diamonds) in the sugar beet region (south: Brooms Barn; north: Kirton). In this YWT network, the min‐max distance between any two traps is 0.29–287 km and the mean 91 km.

Univariate and multivariate spline correlograms were used to estimate the spatial covariance in the weekly and annual *M. persicae* YWT and crop inspection counts as a continuous function of distance for each year and for each week within each year between 2014 and 2020 (Fig. S1a). The R library *ncf* was used to produce spline correlograms and the associated parameters that estimate the strength of spatial synchrony underpinned by a cross correlation approach.^27^ These parameters include the local covariance function (LCF, the value at the intercept on the *Y* axis), the spatial extent, measured in km along the entire length of the *X* axis, and the correlation length (CorL, the value where the spline crosses the *X* axis—zero correlation) measured in km (Fig. 2). Taken together, these three parameters indicate whether spatial synchrony is local or widespread and whether synchrony conforms to theory (*i.e*. that spatial synchrony declines with distance, see Fig. S1a) or opposes theory revealing stochastic local dynamics that produce no such trend.^27^ This approach is relevant because an understanding of spatial synchrony can inform the scale at which sugar beet pests should be managed.

#### 2.4.2 Spatial and temporal generalized additive mixed models

To estimate spatial terms and trends in the YWT and crop inspection data, we used the *mgcv* library for GAMMs alongside the library *gratia*, that provides improved graphic performance for *mgcv*.^28^, ^29^ We fit year and week and produced two types of models: i) a spatial model that included latitude and longitude as Duchon smoothers by the factor YWT sampling week, setting the knots to the maximum to represent detailed local trends, and, ii) a seasonal model that captured the change over YWT sampling weeks for each year using flexible cubic smoothers. Cubic splines (bs = “cr”) were chosen for their flexibility when smoothing time, whereas Duchon splines were chosen to reduce curling (*i.e.*, overfitting) at the edges of spatial boundaries.^28^

All models were fitted using Restricted Maximum Likelihood (REML) assuming the negative binomial distribution for overdispersed data, typical of outbreaking populations. Optimal random effects structures were investigated for each model using the Akaike Information Criterion (AIC) alongside the appraise function for model checking in the *gratia* library. The best performing random effects structure was a simple site random effect term, capturing elements of geography, management and other site specific characteristics. The spatial model computes a random coefficient for both continuous (time, space) and factor variables (week) (Fig. S1a). From these two models we report the significance of the smooth terms that are constrained such that they each sum to a zero mean over the covariate values. As such, the shape of this relationship can be deduced but this does not extend to any systematic difference between temporal or spatial means. Instead, a Wald zero‐effect test is provided to indicate if the smoother is equal to zero (*i.e., p* > 0.05). Significant *p*‐values indicate that the smoother has significantly departed from zero and thus show a non‐zero trend. In the spatial model, parametric coefficients for the week factor estimate differences relative to the first week of responses which naturally follow the seasonal spline, but their use is otherwise limited. To assess model fit, we use the deviance that represents the proportion of the total deviance explained by a model, a goodness‐of‐fit statistic based on the model likelihood.

Discrete‐time autocorrelation models were investigated to examine whether temporal autocorrelation was present within the residuals of the models (Fig. S1a). Using the *gamm* routine that uses penalized quasi‐likelihood, discrete‐time autocorrelation corAR1 parameters were estimated within the linear mixed‐effects model component (LME). Using AIC, the same model with and without the corAR1 term were compared. We also supported LME model interpretation with plots of temporal autocorrelation using normalized residuals to understand if the autocorrelation estimate at a given lag was significantly different from zero.

#### 2.4.3 Forecasts and forecasting error

Historically, in early March each year, pre‐season VY forecasts have been issued by Rothamsted to the sugar beet industry and growers drawing on previous daily suction‐trap counts at Brooms Barn, as shown by Harrington *et al*.^17^ The VY forecasts were driven by predictions of both the first flight and the log_10_ total count to the 17th June that together start the semi‐mechanistic epidemiological model^18^ (Fig. S1b). Predictions were based on a single temperature driver, January–February mean temperature, which has been shown formally to be an important biological driver.^17^, ^21^ The predicted log_10_ total counts for 2002 to 2021 were determined using simple linear regressions that captured the relationships between January–February mean temperatures and the numbers of *M. persicae* caught at Brooms Barn by 17th June each year (Fig. S1b). We evaluate the historical performance of this forecasting model using daily suction‐trap data to calculate the forecasting error (FE). FE is apparent in predicting log total counts (https://repository.rothamsted.ac.uk/), the sole focus of this paper since first flights are well resolved. The difference is derived from the 12.2 m suction‐trap, and the predicted log count to the same date from the linear model (Fig. S1a). Positive FE indicates under‐prediction (*i.e.*, the predicted counts are smaller than the observed values) and negative values indicate over‐prediction, a much rarer event (*i.e*. the predicted counts are larger than the observed values).

#### 2.4.4 Permutation entropy

Permutation entropy (PE) is a model‐free method that was used to provide some insight into the error associated with predicting *M. persicae* abundance. PE quantifies the complexity of a time series and is inversely related to predictability, as shown in Fig. S1c. A refinement of PE that uses normalized weighted ordinal pattern distribution to distinguish between small‐scale noise‐driven variation and large‐scale system‐driven variation, weights each numerical phrase by its variance, producing an entropy value between zero and one.^24^, ^30^ We investigated the frequency of short 3‐day numerical phrases (Fig. S1c) in the R library *statcomp* using daily *M. persicae* counts recorded by the Brooms Barn 12.2 m suction‐trap during the core migration period between standard weeks 19–25 for each year from 2002 to 2021. We then correlate WPE values generated from the Brooms Barn time series with FE, described above, and the proportion of zeros in the time series. By doing so, the degree of FE may be better understood in terms of levels of stochasticity and hence degree of intrinsic predictability.^24^

## 3 RESULTS

### 3.1 Spatial analyses of YWT and crop inspection data

We show that field‐scale spatial synchrony in YWT and crop inspection data is rarely present. Among the univariate year‐week specific correlograms, spatial synchrony was only notable in the 2019 data and only for the first 4 weeks. The synchrony does not extend beyond an average weekly correlation length of 23 km or an average annual correlation length of 60.67 km (Tables 1, 2). The maximum correlation length within any year does not peak in the same week and the local covariance function that would indicate levels of covariance between neighboring traps is often close to zero or at zero, inferring that traps appear to be behaving individually or as a small local cluster (Tables 1, 2). Only in 2019 did YWTs produce a strong spatial synchrony signal, which spanned a maximum distance of 290 km (Fig. 2, Fig. S2). For 2019, the mean annual local covariance function (LCF) at the intercept on the *Y*‐axis is 0.35, higher than for any other year, and the correlation length (CorL) is 89.56 km (Tables 1, 2). Although CorL was not as high as for 2018 (*i.e.*, 127.25 km), for that year the LCF is near zero and the 95% boot‐strapped confidence intervals overlap the zero horizontal threshold, indicating no correlation. The lack of a relationship suggests that the series is dominated by stochasticity with no synchrony (Tables 1, 2, Fig. S2). Apart from 2019, a pattern of no correlation or negative synchrony, indicates that traps further apart are more likely to be spatially covariant than neighboring traps. This pattern was common for both annual and weekly spatial synchrony measures across the YWT network (Tables 1, 2, Fig. 2, Figs. [Link], [Link]). Even after 4 weeks of spatial synchrony, the 2019 YWT weekly counts subsequently decline into no correlation or negative synchrony (Tables 1, 2, Fig. S3).

**TABLE 2:** Combined summary of the generalized additive mixed models and multivariate and univariate spline correlograms for the 2020 crop inspection data

Generalized Additive Model					Spatial Synchrony			
Model type		χ^2^	*p*	Dev	Model type		LCF	CorL
Seasonal model: s(Time)		201.70	<0.001	53%	Annual spatial synchrony		−0.03	0.00
**Spatial model by weeks**		**Χ** ^**2**^	***p***	**Dev** 61%	**Weekly spatial synchrony**		**LCF**	**CorL**
s(Lon, Lat) May 4	Week 17	17.33	<0.05		May 4	Week 17	0.05	31.31
s(Lon, Lat) May 7	Week 18	8.27	0.261		May 7	Week 18	−0.13	0.00
s(Lon, Lat) May 11	Week 18	3.34	0.187		May 11	Week 18	−0.08	0.00
s(Lon, Lat) May 14	Week 19	6.02	<0.05		May 14	Week 19	0.03	84.83
s(Lon, Lat) May 18	Week 19	14.51	<0.001		May 18	Week 19	−0.05	0.00
s(Lon, Lat) May 21	Week 20	19.76	<0.001		May 21	Week 20	0.22	**87.50**
s(Lon, Lat) May 25	Week 20	2.89	0.235		May 25	Week 20	0.04	15.49
s(Lon, Lat) May 28	Week 21	4.93	0.085		May 28	Week 21	−0.07	0.00
s(Lon, Lat) Jun 1	Week 21	0.62	0.734		Jun 1	Week 21	−0.12	0.00
s(Lon, Lat) Jun 4	Week 22	1.05	0.592		Jun 4	Week 22	−0.05	0.00
s(Lon, Lat) Jun 8	Week 22	8.34	0.316		Jun 8	Week 22	−0.05	0.00
s(Lon, Lat) Jun 11	Week 23	0.61	0.738		June 11	Week 23	−0.06	0.00
s(Lon, Lat) Jun 15	Week 23	2.18	0.338		June 15	Week 23	−0.08	0.00

*Note*: “Dev” is the proportion of the total deviance explained by the full model with 61% representing the weekly model deviance explained. LCF is the local covariance function, CorL is the correlation length and the peak in the weekly CorL is highlighted in bold.

**FIGURE 3:**
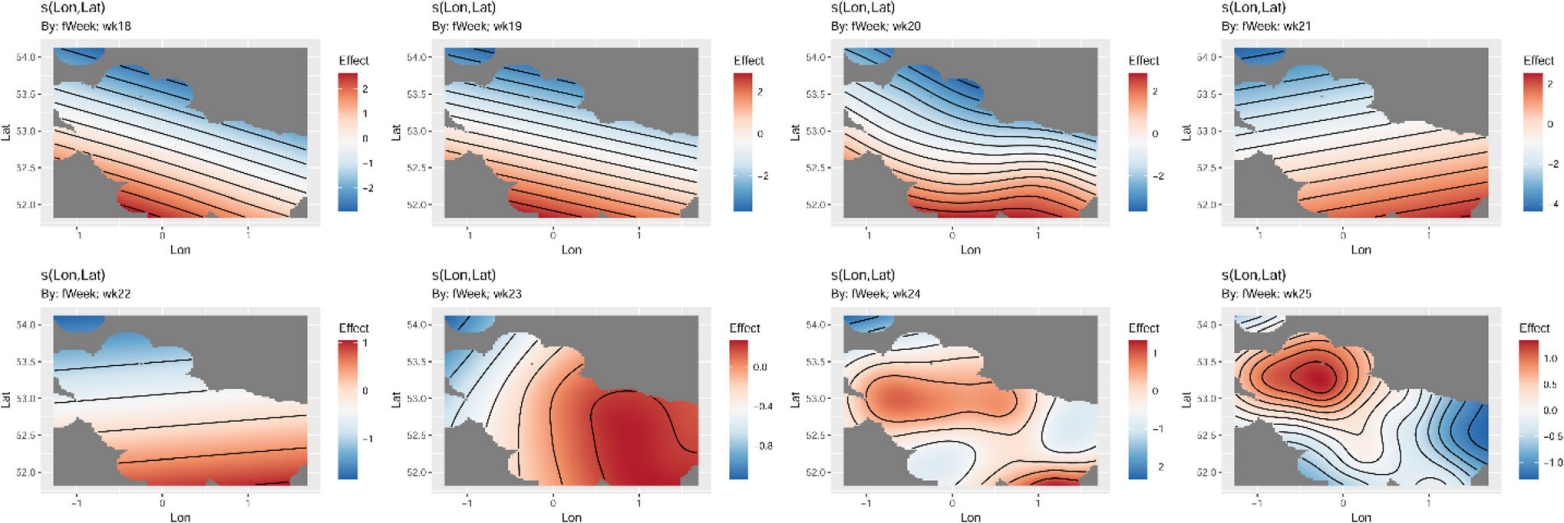
The 2019 detailed results for the generalized additive mixed model of weekly spatial pattern.

There was no common spatial pattern or trend that persisted throughout the time series (Tables 2, 3, Fig. 3, S4). There was a tendency for the season to begin with larger counts in the south (2014, 2015, 2017–2019), although the 2016 and 2020 models showed that these could equally come from the west or the north, respectively (Fig. 3, S4). The total number of spatial terms that are not significant and spatially heterogenous amount to 70% of all the models tested. There were years in which spatial splines did not significantly contribute to explaining any variation in counts across the YWT network for any date in 2014, 2015 and 2018. Generally, there was a pattern for more curvature in the isoclines as the season progressed for all years, indicative of a high level of spatial heterogeneity, further supporting the apparent lack of trend indicated by the synchrony models (Tables 1, 2, Fig. S4).

**TABLE 3:** Summary of the generalized additive mixed models used to generate both a seasonal YWT model to capture the regional population change across sampling weeks and a spatial model to capture detailed YWT local trends

YWT	2014			2015			2016			2017			2018			2019		
Model type	χ^2^	*p*	Dev	χ^2^	*p*	Dev	χ^2^	*p*	Dev	χ^2^	*p*	Dev	χ^2^	*p*	Dev	χ^2^	*p*	Dev
Seasonal model: s(Time)	131.90	<0.001	86%	96.31	<0.001	79%	66.85	<0.001	60.0%	146.10	<0.001	67%	215.05	<0.001	69%	177.20	<0.001	57%
χ^**2**^	**Χ** ^**2**^	***p***	**Dev** 93%	**Χ** ^**2**^	***p***	**Dev** 87%	**Χ** ^**2**^	***p***	**Dev** 74%	**Χ** ^**2**^	***p***	**Dev** 82%	**Χ** ^**2**^	***p***	**Dev** 80%	**Χ** ^**2**^	***p***	**Dev** 76%
s(Lon, Lat) Week 18																32.14	<0.001	***
s(Lon, Lat) Week 19	9.13	0.19					5.67	0.14		4.15	0.13					45.72	<0.001	***
s(Lon, Lat) Week 20	17.14	<0.05					2.65	0.27		2.88	0.24					77.88	<0.001	***
s(Lon, Lat) Week 21	8.85	<0.05		18.15	0.08		0.98	0.61		7.37	<0.05					65.32	<0.001	***
s(Lon, Lat) Week 22	5.41	0.19		7.06	0.20		3.60	0.17		7.92	<0.05		6.40	0.13		12.38	<0.01	**
s(Lon, Lat) Week 23	7.68	<0.05		2.17	0.34		18.33	0.05		12.02	0.05		1.11	0.58		4.61	0.45	
s(Lon, Lat) Week 24	3.56	0.17		17.68	0.08		19.38	<0.05		19.04	0.05		0.55	0.76		27.74	<0.05	*
s(Lon, Lat) Week 25	10.47	0.18		8.10	0.32		0.33	0.33		31.27	<0.01		1.67	0.83		25.20	<0.05	*
s(Lon, Lat) Week 26	18.82	<0.05		4.88	0.52					32.07	<0.01		4.22	0.49				
s(Lon, Lat) Week 27	3.38	0.18		6.76	0.12					22.67	<0.05		1.32	0.52				
s(Lon, Lat) Week 28													9.03	0.35				

*Note*: The total number of significant spatial terms (s(Lat, Lon)) that are not significant and spatially heterogenous amount to 70% of the time series. “Dev” is the proportion of the total deviance explained by the full model with 74%–93% representing the range of deviance explained values for the weekly models. Grey boxes indicate data were too sparse to run a GAMM.

### 3.2 Within‐season model

**FIGURE 4:**
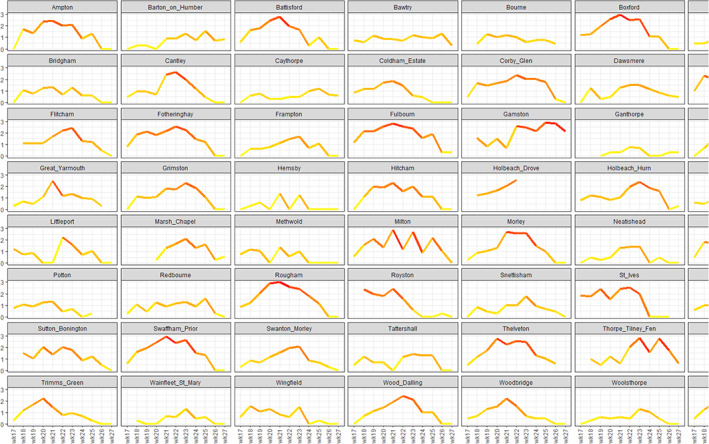
The *Y*‐axis depicts the log_10_ counts of *Myzus persicae* as recorded by YWTs between weeks 17 and 27 during 2019, where high counts are recorded in red and relatively low counts recorded in yellow. The plots show the generally low or zero values in week 17 and the subsequent population collapse after week 25 at most sites.

The seasonal model indicated that there was no one typical season that took a common form, either peaking in the same week or having the same shaped spline (Fig. S5). Within 2019, logged counts showed considerable variation and that is typical of all years (Fig. 4). There is a tendency for *Myzus* numbers to accumulate for the first 3 weeks, reaching a peak soon after, though this is not universal (Fig. 4). The 2019 seasonal GAMM suggests that there may be two peaks on average in the 4th and 6th weeks followed by a decline in numbers (Fig. S5).

We found extremely weak evidence for temporal autocorrelation with a tendency to produce inflated AIC values when corAR1 was included (*i.e.*, 2015–2017 and 2019 Table S1). Although the AIC was smaller when a corAR1 process was included in 2014 and 2018, lag‐wise 95% confidence intervals were not exceeded when the corAR1 term was absent from the model. In 2015, a marginal autocorrelation of lag term at 7 weeks, effectively the entire length of the season, was stronger when the corAR1 term was included, and the model term was dismissed. In 2020, AIC indicated that when a corAR1 process was included models performed better. Temporal autocorrelation appeared for 3 weeks in the normalized residuals ACF plot, although this lag marginally overlapped 95% confidence intervals (Table S1). However, when a corAR1 process was included, the corAR1 term introduced a much stronger lag of 2 weeks and a stronger lag term of 3 weeks compared to a model without corAR1. Consequently, the corAR1 term was dropped from all models.

### 3.3 Forecasts and forecasting error

**FIGURE 5:**
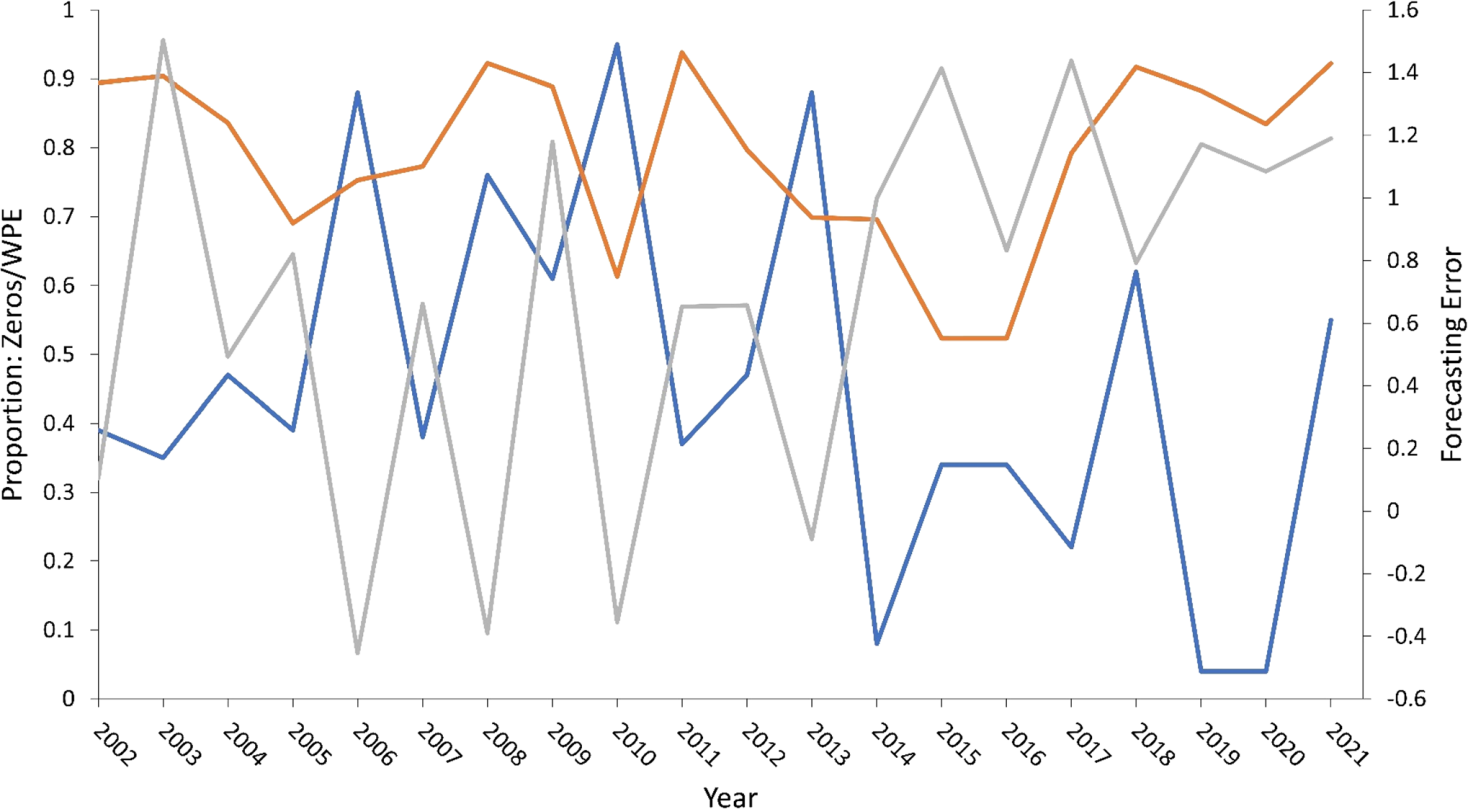
Weighted permutation entropy and forecasting error (FE) for *Myzus persicae* derived from the Brooms Barn 12.2 m suction‐trap during the core migration period (weeks 19–25 for each year) from 2002 to 2021. Short 3‐day numerical phrases are colored orange and the proportion of zeros is colored blue, and both are represented by Y1 axis. FE (Y2 axis), colored grey, is the difference between the log_10_ observed counts derived from the 12.2 m suction‐trap to the 17th June and the log_10_ counts to the 17th June that were predicted from simple linear regression models. Generally, there is a consistent under‐prediction of log_10_ counts, apart from 2006, 2008 and 2010 and marginally in 2013 when the linear model over‐predicted (*i.e*. negative FE: predicted counts exceeded observed values), see the patterns of accumulation each year (Fig. S1b).

Short 3‐day numerical phrases had weighted permutation entropy (WPE) values that ranged from 0.527 to 0.938, indicating that these time series have moderate to low intrinsic predictability and hence moderate to high stochasticity. There was weak correlation between FE and 3‐day numerical phrases (Spearman rank correlation, *r*_s_ = 0.435, *p* = 0.055). The highest FE around 1.5 indicate under prediction, and are associated with a wide range of “proportion of zero” values (0.52–0.90). This underpins the relatively weak correlation coefficient. However, the correlation between FE and the “proportion of zeros” is strongly negative (*r*_s_ = −0.658, *p* = 0.001), indicating that at high FE values, the “proportion of zeros” is very low. Figure 5 shows that the FE does not closely follow the shape of the short 3‐day WPE phrases. When permutation entropy produces high WPE values (*e.g.*, 2008 = 0.992, 2011 = 0.938, 2021 = 0.922), inferring lower intrinsic predictability, FE is wide ranging (2008 = −0.390, 2011 = 0.653, 2021 = 1.190). Furthermore, Fig. 5 does not show a regime shift after the withdrawal of neonicotinoids in 2018. Although WPE values are high (*i.e.*, 2018 = 0.917, 2019 = 0.883, 2020 = 0.834, 2021 = 0.922), these values alone are not exceptional (*cf*. 2008, 2011). However, taken together the series of 2018–2021 WPE values is notable and perhaps indicative of heterogeneity in the management and incidence of aphids.

## 4 DISCUSSION

### 4.1 Spatial ecology of *M. persicae*

We show little evidence of spatial synchrony in weekly or annual counts of *M. persicae* and only weak effects of latitude and longitude. Specifically, spatial synchrony was rarely present and did not persist for more than 4 weeks or extend beyond an average weekly correlation length of 23 km or an average annual correlation length of 60 km. Instead, without temporal autocorrelation, local spatial heterogeneity increases as the field season progresses. Only in 2019 did statistically significant spatial terms and an overall spatial synchrony pattern emerge, largely as a result of strong spatial synchrony in the first 4 weeks, that extended to 90 km. In summary, these results indicate the unpredictability of aphid field counts across the region, that limit regionwide strategies for control.

A general lack of synchrony contrasts with previous studies using 12.2 m suction‐traps.^19^, ^20^, ^22^, ^27^, ^31^, ^32^ These studies are compelling but raise the question as to why our results showed contrasting and asynchronous population fluctuations. Large‐scale synchrony does not necessarily imply that the aphids at the field level are behaving similarly. We argue that because the scale of the region under study was small (*i.e*. <300 km) compared to previous synchrony studies working at the national scale and beyond (>800 km), weather variation would be unlikely to explain local dynamics. Weather patterns did undoubtedly differ and this may be most severely felt in the winter, when *M. persicae* would likely suffer late winter frosts.^23^ However, during the period of our study temperature did not present a limiting factor; the YWT season started in May when days of ground frost rarely exceeded 10 days in total and the mean monthly temperature was at least 10 °C, well above *M. persicae*'s lower walking temperature threshold of 4 °C.^33^, ^34^ At approximately 15–16 °C, *M. persicae* will take flight and these conditions were met in our study from May onwards when mean daily maximum temperatures indicated that this threshold was easily reached.^34^, ^35^, ^36^ Other meteorological factors may be more important than temperature alone. Behavioral responses to wind conditions during flight are likely to explain variation and potentially lack of synchrony between YWTs. Wind speed and direction is very difficult to capture at the field scale, though we know that behaviors termed “appetitive” flight over distances of less than 100 m are strongly correlated with both wind direction and speed.^37^ Close to the ground and below wind speeds of 0.15 m/s, *M. persicae* will perform station keeping behaviors – angular flight paths with seemingly random turns.^38^ Should wind speeds exceed flight speeds that are estimated to be ≈0.41 m/s, individuals will fly downwind contributing little to the direction and speed.^37^, ^38^, ^39^ Above 1 m/s, aphid flight becomes increasingly rarely observed near the ground.

Each YWT was placed in a sugar beet field, one of the major host plant associations of *M. persicae*, and thus the feeding niche was suitable and uniformly available across the trap network. However, one major spring dispersal pathway into sugar beet is *via* winter oilseed rape, a winter host that could provide a green bridge into sugar beet and contribute local variation depending on its availability. Cocu *et al*.^20^ showed that oilseed rape was the only land use variable to link to *M. persicae* populations which explained 18% of the variation. However, the highly polyphagous nature of *M. persicae* which has over 40 plant families (>200 sp) within its host plant range, may also explain differences between YWT catches.^13^, ^14^ Most agricultural “weeds” are hosts^16^, ^40^, ^41^ and whilst some are widely distributed at the 1 km‐grid scale, such as the cosmopolitan Shepherd's Purse, *C. bursa‐pastoris*, others are much more patchy (*e.g.*, Corn spurry, *Spergula arvensis* L.) hence introducing host plant spatial heterogeneity.^15^ Beet clamps and weed beet that persist in the field are also hosts and virus reservoirs,^35^ and whilst much has been done to reduce these vector sources as part of a program of better crop hygiene measures, local variation in weed beet and clamp management may explain differences between YWT catches.^12^ Other factors that could contribute local variation were trap type and height, but these were standardized *a priori* and background contrast ratios between soil and trap were broadly uniform given that most sugar beet growers drill their seed at a similar time.^42^

### 4.2 Predictability of *M. persicae* numbers

To understand population change and evaluate our forecasting approach, we used weighted permutation entropy, a model free method to estimate time series complexity, intrinsic predictability and levels of stochasticity.^24^ Overall, WPE indicates that *M. persicae* time series are driven by stochastic forcing, particularly when counts are high and zeros low, though different WPE values can be associated with the same final accumulated count (Fig. S1b). The stochastic component reduces the intrinsic predictability and arises due to direct and indirect factors acting on each individual aphid, but not it seems due to a regime change caused by the withdrawal of neonicotinoids in 2018. These factors include, but are not limited to, developmental time to wing production, driven by temperature, the conditions at take‐off, driven by wind, light intensity, host plant type and quality amongst other behavioral and atmospheric factors, particularly a temperature flight threshold of 16–17 °C.^36^, ^43^ Another stochastic element, the probability of capture is not well understood^42^ and is likely a function of the aforementioned above, and physiological factors including fat reserves and exhaustion, as well as the strength of atmospheric convection.^44^

### 4.3 Forecasting models: shortfalls and improvements

Whilst climate change is driving aphid migrations earlier, first flights are easily predicted. However, there remains large scale variation in log abundance over time.^45^ Our results show that simple linear regressions tend to under‐predict log_10_ counts to the 17th June. It is clear that very short timescale population changes are poorly explaining this pattern. However, within six generations, 318 750 000 descendants could be produced from just one aphid, hence challenging any model.^46^

Our regression model could potentially be improved by including a stochastic component, as suggested by Kindlmann and Dixon,^47^ but predictions are then themselves stochastic. Small catches at the start of the season are difficult to estimate and the conditions during early population growth are likely to be critical when estimating abundance. This complexity may instead favor the use of artificial neural networks (ANNs), though a successful outcome is dependent on a large training dataset. Perhaps one of the most successful aphid models has been for the grain aphid, *Sitobion avenae*.^48^ However, we doubt whether even this model could predict the fine scale spatial heterogeneity of YWT *M. persicae* weekly counts, as captured by our mixed models.

## CONFLICT OF INTEREST DECLARATION

The authors declare no conflict of interest.

## AUTHOR CONTRIBUTIONS

SJC prepared the data for analysis, implemented the linear regressions and produced the linear regression forecasts. JRB implemented all remaining analyses and wrote the manuscript. All authors provided comments on the analyses and manuscript and approved the manuscript. BBRO provided yellow water trap data.

## Supporting information


**Figures S1a,b,c:** Spatial Analysis, Forecasting Error and Permutation Entropy

**Figures S2:** Annual Spatial Synchrony Models

**Figures S3:** Weekly Spatial Synchrony Models

**Figures S4:** Spatial Generalized Additive Mixed Models

**Figures S5:** Seasonal Generalized Additive Mixed Models

**Table S1.** Mixed Model Autocorrelation Function corAR1

## Data Availability

The data that support the findings of this study are openly available in Rothamsted Repository at https://repository.rothamsted.ac.uk/.
