# Supplementary material for: Quantifying inherent predictability and spatial synchrony in the aphid vector Myzus persicae: field‐scale patterns of abundance and regional forecasting error in the UK
Source: Pest Manag Sci. 2022 Dec 19;79(4):1331–41. doi: 10.1002/ps.7292 (PMC10952309; doi:10.1002/ps.7292)
Supplement: Supplementary file 1 — Figures S1a,b,c: Spatial Analysis, Forecasting Error and Permutation Entropy [file PS-79-1331-s004.pdf]

Supplementary Materials S1

S1a: Spatial Analyses

S1b: Forecasting Error

S1c: Permutation Entropy

S1a:Spatial Analyses Spatial Synchrony & Generalized Additive Mixed Models

The spline correlogram provides:

- A direct estimate of the covariance function as a continuous function of distance, based on site-specific latitude and longitude, using a local averaging step function.
- 95% boot-strapped confidence intervals.
- The extent of local synchrony along the *X* axis in km where it dissects the line
- A measure of the local covariance function at the intercept on the *Y* axis
- See https://cran.r-project.org/web/packages/ncf/ncf.pdf for a description and https://github.com/objornstad/ncf/blob/master/R/spline-correlog.R for the code.

There are two forms of the spline correlogram:

- Univariate – the analysis is conducted on a vector representing a single week using a step function. This step function is a method for calculating the local spatial covariance across a range of focal distances, to then approximate its continuous function over the entire spatial extent as a spline. The bandwidth parameter *h* adjusts the smoothness of the spline based on those local distances, that itself is determined by the distance class width parameter λ. The function uses the scalar product on Euclidean distances, a 2-dimensional calculation for parallelism. The overall spatial dependence is measured by Moran's *I*.

- Multivariate – as above but the analysis is conducted on a set of vectors, representing multiple sampling weeks that have identical dimensions, to estimate a cross-correlogram. These correlograms are first differenced time series of abundance (i.e. using the difference between successive observations). In essence, this is a cross-product statistic, a 3-dimensional calculation that reports the size of the parallelism in Euclidean space between a variable and its spatial lags, with the variable expressed in deviations from its mean. The smooth is on the (lower.tri + diagonal) and centred (default) Pearson correlation matrix on distance. The overall spatial dependence is measured by a centred Mantel statistic.

Bjornstad, O.N., Ims, R.A. & Lambin, X. (1999) Spatial population dynamics: Analysing patterns and processes of population synchrony. Trends in Ecology and Evolution, 11, 427-431.

Bjornstad, O.N. & Falck, W. (2001) Nonparametric spatial covariance functions: estimation and testing. Environmental and Ecological Statistics, 8:53-70. https://doi.org/10.1023/A:1009601932481

Spatial Synchrony Theory: Covariance

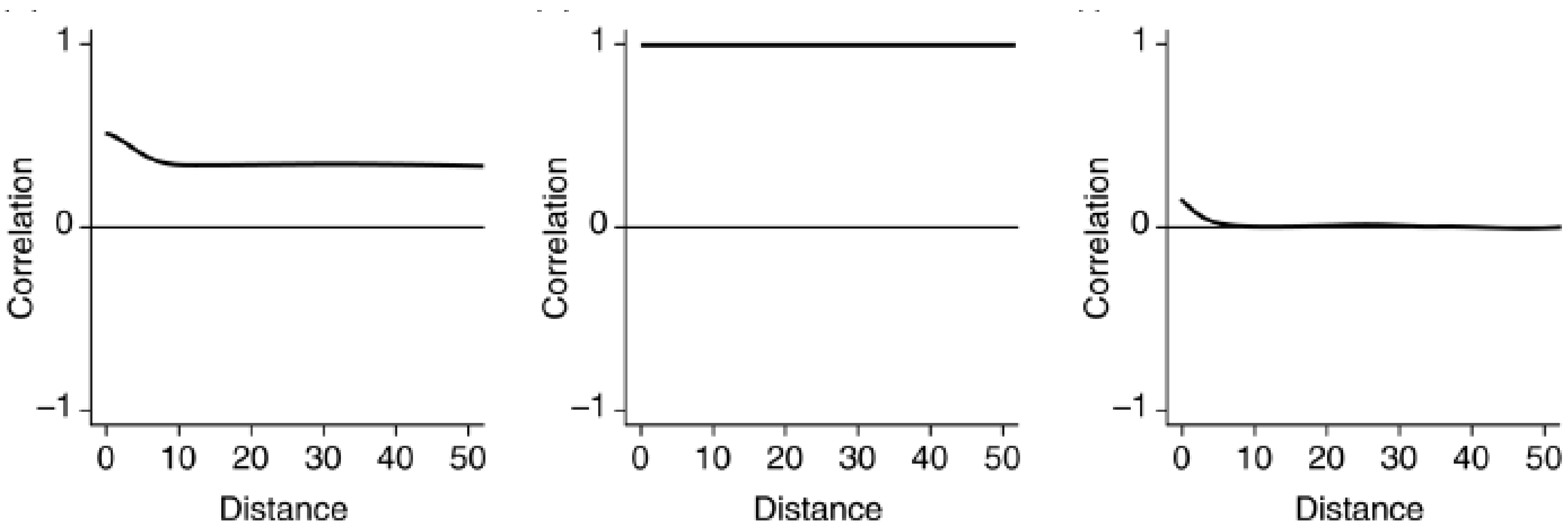

Local covariance is moderate
Decays to regional synchrony
over entire distance.

Local covariance is high
and phase-locked aka coupled
over entire distance. Perfect
synchrony

Local covariance is near zero
Soon it is stochastic with no
synchrony

Bjornstad, O.N., Ims, R.A. & Lambin, X. (1999) Spatial population dynamics: Analysing patterns and processes of population synchrony. Trends in Ecology and Evolution, 11, 427-431.

Example Spatial Synchrony R code

```
library(ncf)
setwd("")
YWT19<- read.table("data.csv", header = TRUE, sep =",")
attach (YWT19)
#format of "YWT2019.csv"
```

Site	Lat	Lon	wk18	wk19	wk20	wk21	wk22	wk23	wk24	wk25
Ampton	52.28852	0.75004	51	22	225	266	103	125	7	21
Barton_or	53.67603	-0.40755	1	1	0	7	7	20	5	34
Battisford	52.14999	0.952596	40	65	289	616	93	48	1	9
Bawtry	53.44705	-1.04537	3	13	7	6	4	14	9	8
Bourne	52.74344	-0.26741	2	18	10	14	9	3	5	5

```
x= YWT19$Lon
y= YWT19$Lat
# multivariate
#run the synchrony analysis across all weeks for 2019
#combine weeks into a dataframe
sam.week<-data.frame(cbind(wk18,wk19,wk20,wk21,wk22,wk23,wk24,wk25))
splinecorrelcount.2019.wk18.to.wk25<- spline.correlog(x=x, y=y, z= sam.week, resamp =1000, latlon = TRUE, na.rm = T)
summary (splinecorrelcount.2019.wk18.to.wk25)
#estimate
estimate x= 89.56138 e= NA y =0.3559117
plot (splinecorrelcount.2019.wk18.to.wk25, main = "YWT 2019 Weeks 18-25. Spatial extent =  90 km" )

#univariate
# now generate a weekly model, using individual week columns within sam.week
# use the first week as an example, but continue computing parameters for subsequent weeks (not # shown)
# By week. From 18 to …
splinecorrelcount.2019.wk18 <- spline.correlog(x=x, y=y, z= sam.week[, 1], resamp =1000, latlon = TRUE, na.rm = T)
summary (splinecorrelcount.2019.wk18)
#estimate
estimate x=48.2477 e=14.51873 y=0.5026474
plot (splinecorrelcount.2019.wk18, main = "YWT 2019 Week 18. LCF =  48 km")
etc
```

Example GAMM R code

```
setwd("")
YWT.2019.gam<- read.table(“data.csv", header = TRUE, sep ="," )
attach (YWT.2019.gam)
# format for mgcv long format. The … indicates more sites were recorded but removed to show week 19 data
```

Week	Time	Site	Lat	Lon	Myzus
wk18	1	Ampton	52.28852	0.75004	51
wk18	1	Barton_or	53.67603	-0.40755	1
wk18	1	Battisford	52.14999	0.952596	40
wk18	1	Bawtry	53.44705	-1.04537	3
wk18	1	Bourne	52.74344	-0.26741	2
……					
wk19	2	Ampton	52.28852	0.75004	22
wk19	2	Barton_or	53.67603	-0.40755	1
wk19	2	Battisford	52.14999	0.952596	65
wk19	2	Bawtry	53.44705	-1.04537	13
wk19	2	Bourne	52.74344	-0.26741	18

```
library (mgcv)
# Gavin Simpson's parallel package which provides better spatial plots for mgcv models for plot() use as
# 'draw(model)' see here
library(gratia)
fsite <- as.factor(Site)
fWeek <- as.factor(Week)
```

1.

```
# simple model of the whole season with a random effect for site
# REML penalises wiggliness and produces better resolved models compared to GCV
# k is the number of knots (i.e. determining the splines wiggliness)
season.gam.1<- gam(Myzus~ s(Time, bs="cr", k=8)+ s(fsite, bs="re"),
family=nb, method = 'REML', data= YWT.2019.gam)
draw (season.gam)
appraise(season.gam)
```

Example GAMM R code

2.

```
# seasonal gamm with temporal autocorrelation
# https://fromthebottomoftheheap.net/2014/05/09/modelling-seasonal-data-with-gam/
# gamm uses nlme framework as part of correlated autoregressive model. call LME #component.
# ~1 random intercept for each site
#corAR1() works with discrete time.
season.gam.nb.corAR1<- gamm(Myzus~ s(Time, bs="cr", k=8), random=list(Site=~1), family=nb, method
= 'REML', correlation = corAR1(), data= YWT.2019.gam)
#The default for `resid()`won't take the covariance matrix into account. The normalised residuals will
#do that:
# with corAR1
layout(matrix(1:2, ncol = 2))
acf(resid(season.gam.nb.corAR1$lme, type ="normalized"), main = "acf season.gam.nb.corAR1")
pacf(resid(season.gam.nb.corAR1$lme, type ="normalized"), main = "pacf season.gam.nb.corAR1")
```

3.

```
#seasonal gamm spatial with temporally independent model
# more detailed model of the season by week using a factor smooth approach
# y ~ f+ s(x, by =f). Separate smooth functions for each level of the factor which is centred    # and does
not include the group means. Must include f, the factor separately because
# parametric term f includes the uncentred means which becomes testable.
# Duchon splines bs ='ds' cope better with the spatial boundaries of a model, preventing       # curling at
the edges
spatialgam.week<- gam(Myzus~ fWeek+ s(Lon, Lat, bs= 'ds', by=fWeek, k=32)+ s(fsite,bs="re"), family = nb,
method = 'REML', data = YWT.2019.gam)
draw(spatialgam.week)
summary (spatialgam.week)
```

S1b: Forecasting Error

- In a retrospective study, we used simple linear regression to predict the number of aphids caught in the Broom's Barn suction-trap to 17th June from average daily mean temperatures over the 59-day period 1st January to 28th February inclusive.

- Abundances were predicted for each of 2002 to 2021 in turn, with each regression including abundance and temperature data from 1965 up to the year previous to the prediction year (so N = 37…55 for 2002…2021, respectively). In this way the most up to date aphid information was used to produce each subsequent forecast.

- All observed counts were logged (base 10 after adding an offset of unity to cope with zeros) before analysis. The adjusted coefficient of determination (r^2_{adj}) ranged between 0.552 (2019) and 0.664 (2003).

- For each prediction year, the forecasting error was defined as the difference between the observed log count to the 17th June, derived from the 12.2 m suction-trap, and the predicted log count to the same date, derived from the linear model (Figure S1a).

FIGURE S1a

Predicted and observed counts of *M. persicae* to the 17th June (log scale, base 10) for the Broom's Barn 12.2 m suction-trap from 2002 (02) to 2021 (21). Predictions were derived from a simple linear regression model relating observed counts to average January-February daily mean temperatures.

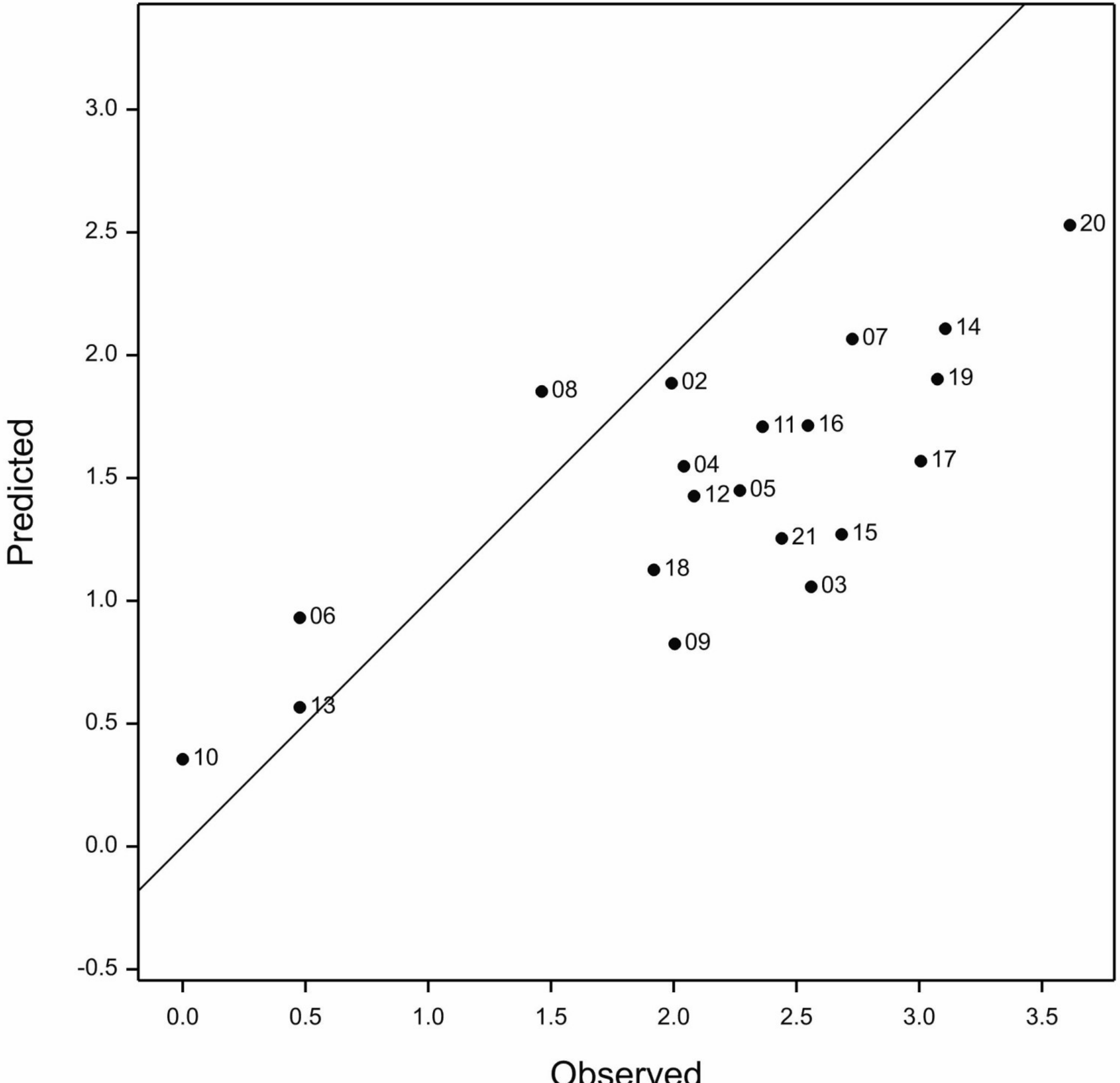

TABLE S1a. Summary of results from the retrospective forecasting error study. Observed and predicted counts of *M. persicae* to 17th June are on $\log_{10}$ scale (after adding an offset of unity). Forecast error is the difference between the observed and predicted logged counts. Intercept and slope are the parameters of simple linear regressions relating historical *M. persicae* counts to 17th June to average January-February daily mean temperatures.

Prediction year	Observed	Predicted	SE(Predicted)	Forecast error	Intercept	SE(Intercept)	Slope	SE(Slope)	r^2_{adj} (%)
2002	1.99	1.89	0.128	0.11	-0.48	0.195	0.391	0.048	64.7
2003	2.56	1.06	0.069	1.50	-0.49	0.190	0.394	0.046	66.4
2004	2.04	1.55	0.100	0.49	-0.45	0.217	0.395	0.052	59.6
2005	2.27	1.45	0.089	0.82	-0.47	0.216	0.402	0.052	60.2
2006	0.48	0.93	0.081	-0.45	-0.48	0.221	0.410	0.053	59.7
2007	2.73	2.07	0.144	0.66	-0.50	0.220	0.412	0.053	59.5
2008	1.46	1.85	0.114	-0.39	-0.55	0.218	0.428	0.052	61.8
2009	2.00	0.82	0.085	1.18	-0.53	0.216	0.421	0.051	61.4
2010	0.00	0.36	0.131	-0.36	-0.47	0.225	0.412	0.053	57.5
2011	2.36	1.71	0.103	0.65	-0.50	0.217	0.419	0.052	59.0
2012	2.08	1.43	0.082	0.66	-0.52	0.218	0.427	0.051	59.6
2013	0.48	0.57	0.107	-0.09	-0.52	0.219	0.431	0.052	59.3
2014	3.11	2.11	0.131	1.00	-0.53	0.214	0.432	0.051	59.9
2015	2.68	1.27	0.075	1.41	-0.58	0.217	0.451	0.051	61.3
2016	2.55	1.71	0.096	0.83	-0.56	0.229	0.453	0.054	58.2
2017	3.01	1.57	0.086	1.44	-0.58	0.232	0.460	0.054	58.3
2018	1.92	1.13	0.085	0.79	-0.58	0.243	0.469	0.057	56.3
2019	3.07	1.90	0.109	1.17	-0.56	0.244	0.466	0.057	55.2
2020	3.61	2.53	0.165	1.08	-0.58	0.247	0.477	0.058	55.5
2021	2.44	1.25	0.085	1.19	-0.66	0.250	0.501	0.058	57.5

FIGURE S1b

Accumulation of *M. persicae* caught at Brooms Barn between late March and end August each year from 2002-2021.

x-axis: Day number in year

(scale = 82 – 243)

y-axis: Log_{10}-transformed accumulated count

Year and day number of first flight given in headings

NB: Count on day 103 in 2020 was from a 7-day catch; first flight estimated to be day 100 for analyses/forecasts

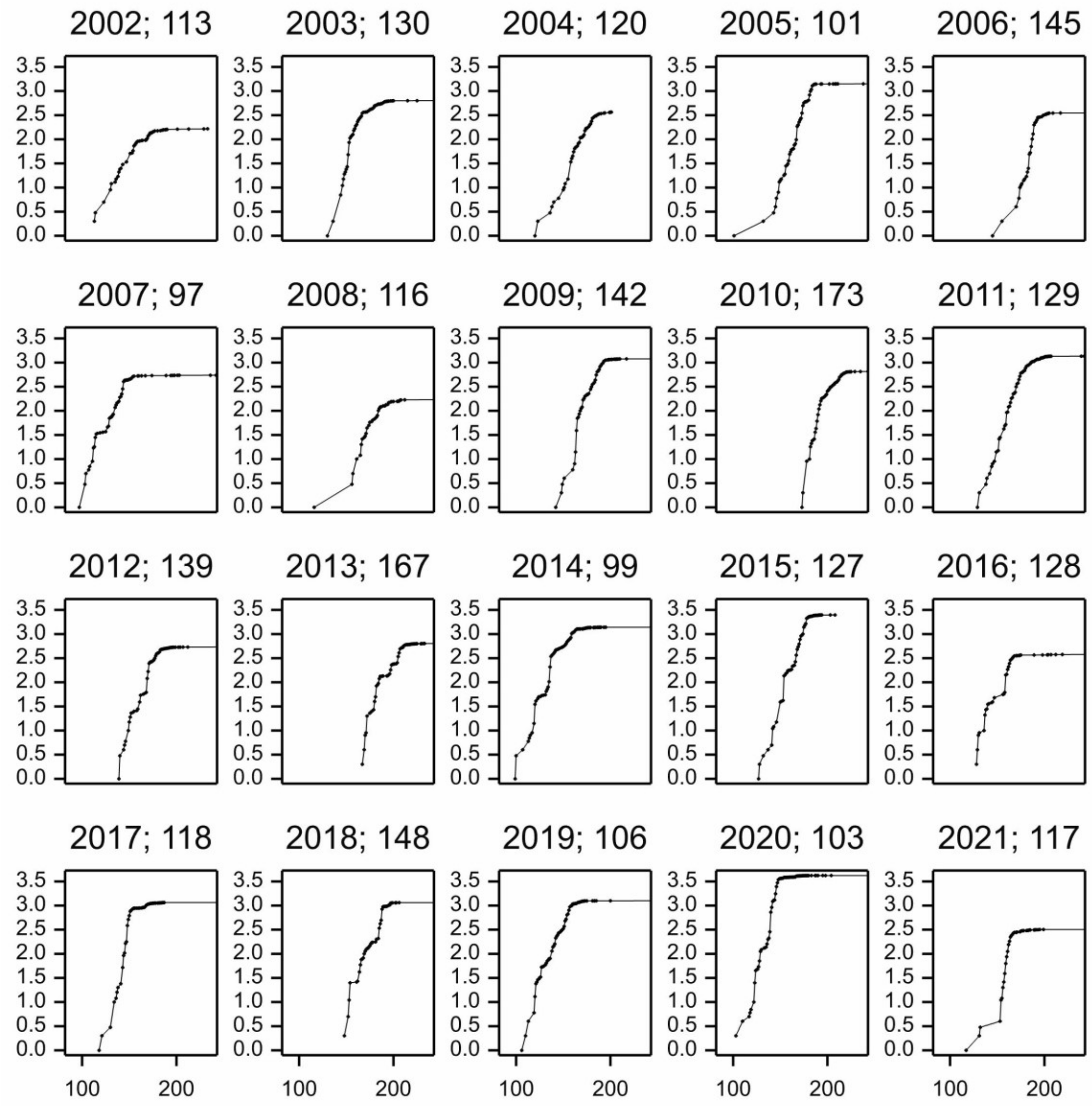

S1c Permutation Entropy

- Entropy has a perfect inverse relationship with knowledge, where the more knowledge there is, the lower the entropy and the easier it is to produce a prediction for the system
- Permutation entropy (PE) is a type of entropy measure
- Model free method approximating the rate at which new information is generated along a time series and how this is transmitted from past states to the present.

Pennekamp et al. (2019) The intrinsic predictability of ecological time series and its potential to guide forecasting. *Ecological Monographs* 89(2):e01359. 10.1002/ecm.1359

Define a window length, that slides along a time series.

- In our example, the window length (m) is short (3days) numerical phrase is being sought using ordinal ranks.
- If *m*=3 and *t*=1, then the phrase 110(x_1),42 (x_2),119 (x_3) is converted into ordinal ranks as 42 (x_2),110 (x_1),119(x_3) yielding **2,1,3** number sequence.
- It will permute or slide the window down 1 to the next phrase ….. $x_{3,}$ x_1 x_2 , which yields **3,1,2** number sequence etc
- The frequency distribution of these phrases then allows a measure of the stochastic/deterministic components.
- library(statcomp) in R the function weighted_ordinal_pattern_distribution(x=x, ndemb = 3) were used to computed unweighted and weight PE respectively.
- An offset of 1 was added to allow for zeros

Broom's Barn 2019

June1	110	
June2	42	**2,1,3**
June3	119	
June4	30	
June5	130	

June1	110	
June2	42	
June3	119	**3,1,2**
June4	30	
June5	130	
