# Supplementary figures and images for: Quantifying inherent predictability and spatial synchrony in the aphid vector Myzus persicae: field‐scale patterns of abundance and regional forecasting error in the UK
Source: Pest Manag Sci. 2022 Dec 19;79(4):1331–41. doi: 10.1002/ps.7292 (PMC10952309; doi:10.1002/ps.7292)

# Multivariate Annual Spline Correlograms YWT 2014-2019 and Crop Inspection Data 2020

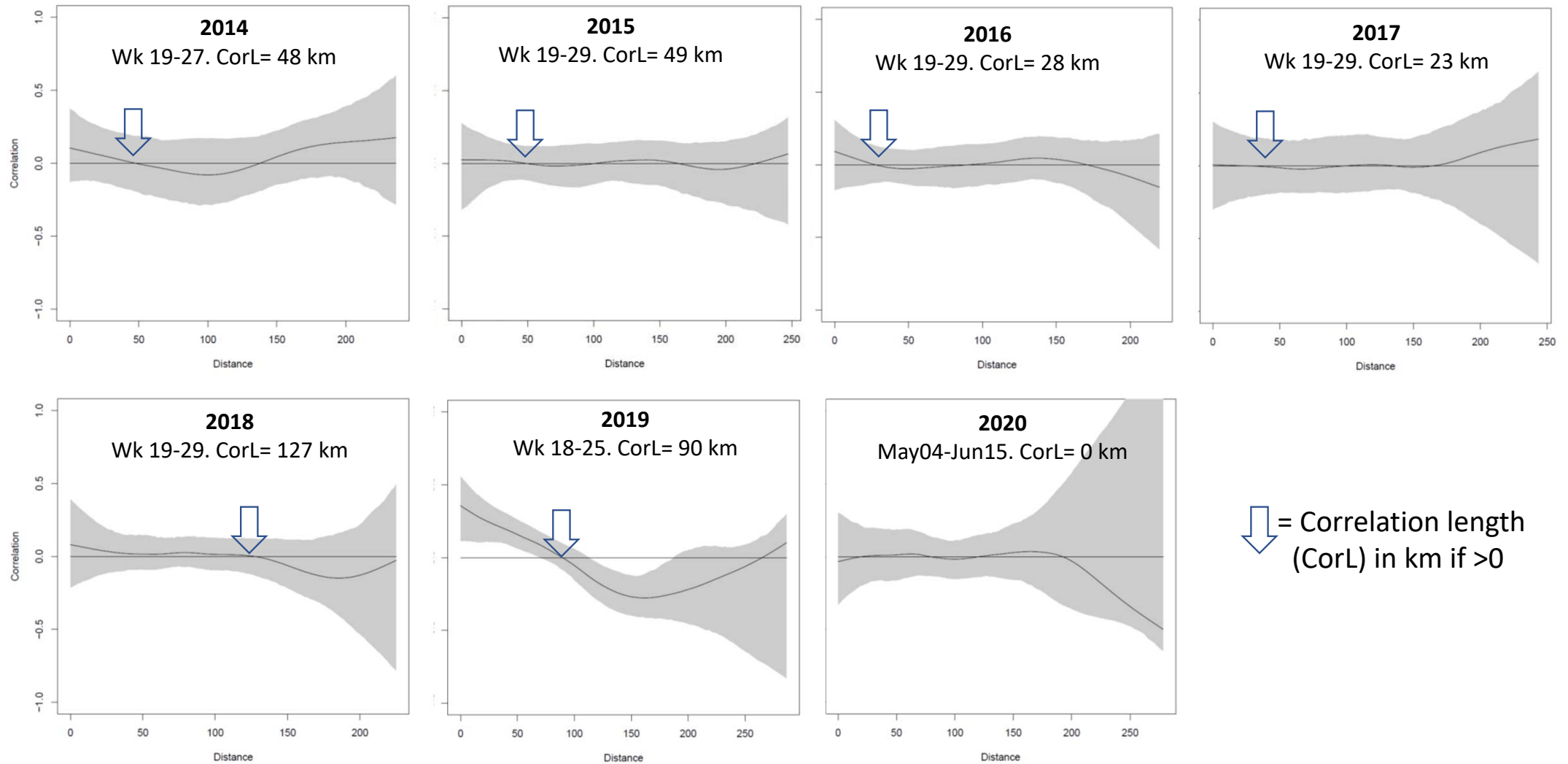

Supplement: Supplementary file 2 — Figures S2: Annual Spatial Synchrony Models [file PS-79-1331-s003.pdf]

# Seasonal Generalized Additive Mixed Models YWT 2014-2019 and Crop Inspection Data 2020

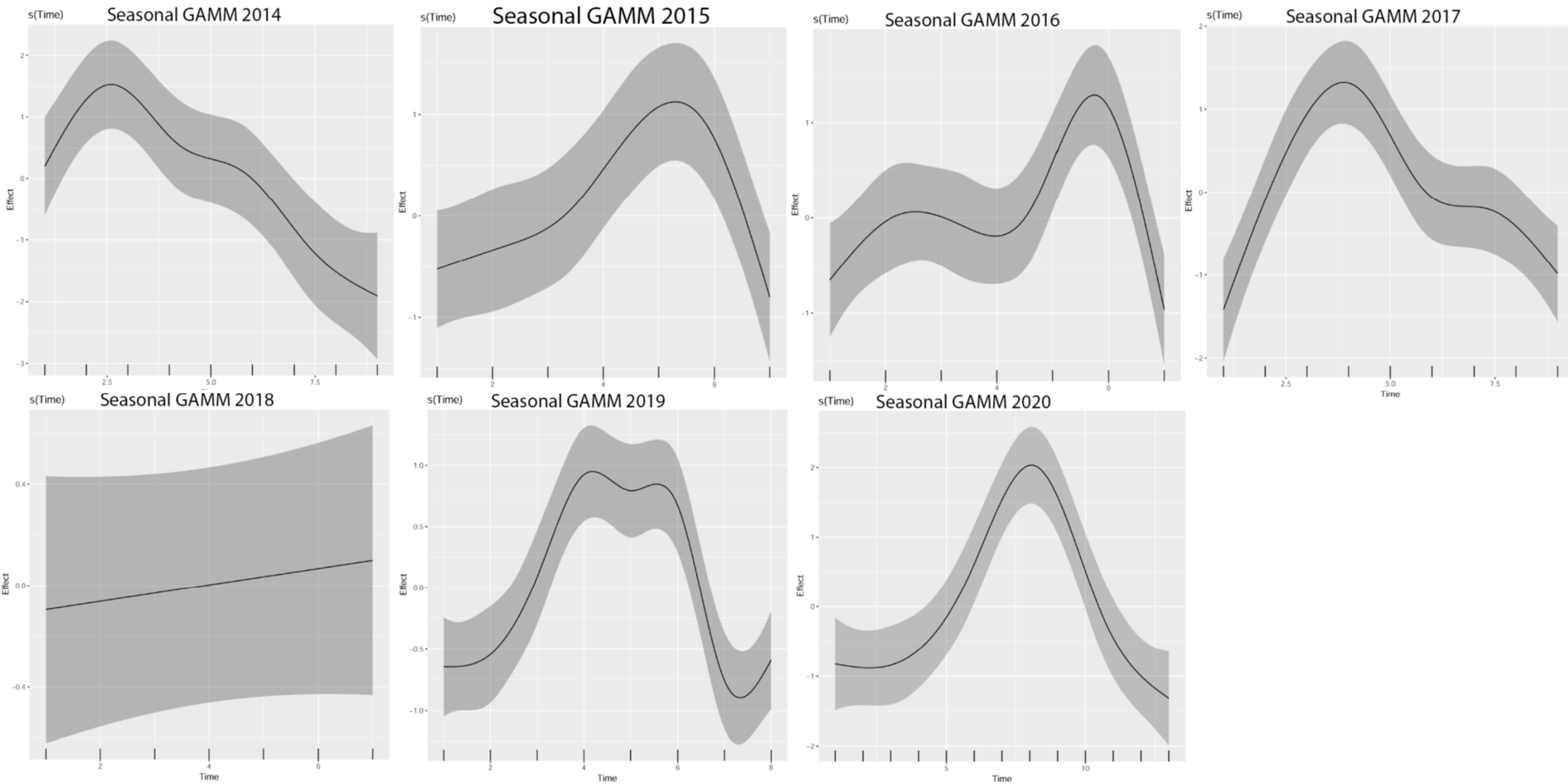

Supplement: Supplementary file 5 — Figures S5: Seasonal Generalized Additive Mixed Models [file PS-79-1331-s001.pdf]
