# Supplementary material for: Quantifying inherent predictability and spatial synchrony in the aphid vector Myzus persicae: field‐scale patterns of abundance and regional forecasting error in the UK
Source: Pest Manag Sci. 2022 Dec 19;79(4):1331–41. doi: 10.1002/ps.7292 (PMC10952309; doi:10.1002/ps.7292)
Supplement: Supplementary file 3 — Figures S3: Weekly Spatial Synchrony Models [file PS-79-1331-s005.pdf]

Univariate Weekly Spline Correlograms
YWT 2014-2019 and Crop Inspection Data 2020

= Correlation length
(CorL) in km if >0

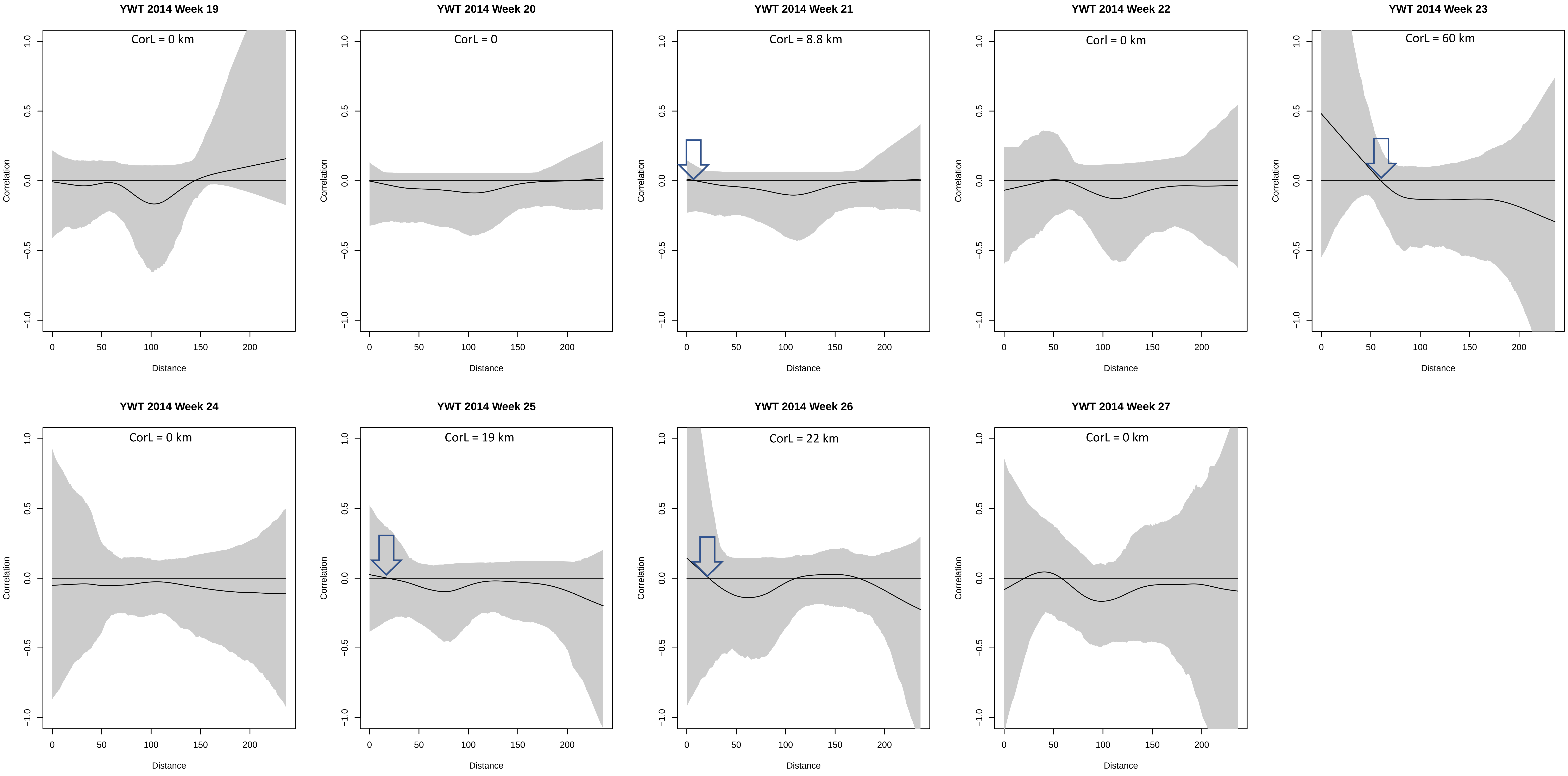

YWT 2014 Week 19
CorL = 0 km
YWT 2014 Week 20
CorL = 0
YWT 2014 Week 21
CorL = 8.8 km
YWT 2014 Week 22
Corl = 0 km
YWT 2014 Week 23
CorL = 60 km
YWT 2014 Week 24
CorL = 0 km
YWT 2014 Week 25
CorL = 19 km
YWT 2014 Week 26
CorL = 22 km
YWT 2014 Week 27
CorL = 0 km
Correlation
Distance

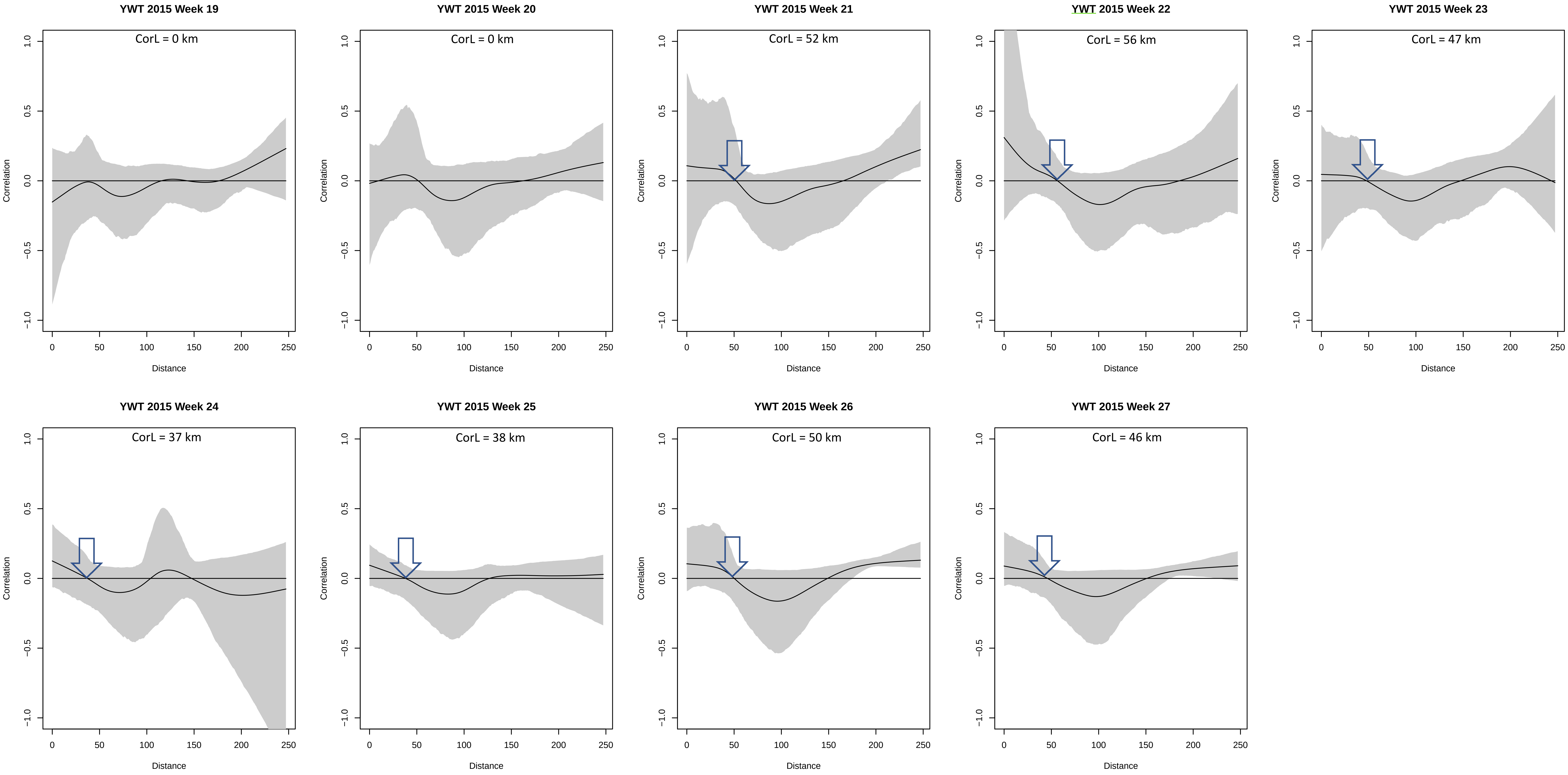
YWT 2015 Week 19
CorL = 0 km
YWT 2015 Week 20
CorL = 0 km
YWT 2015 Week 21
CorL = 52 km
YWT 2015 Week 22
CorL = 56 km
YWT 2015 Week 23
CorL = 47 km
YWT 2015 Week 24
CorL = 37 km
YWT 2015 Week 25
CorL = 38 km
YWT 2015 Week 26
CorL = 50 km
YWT 2015 Week 27
CorL = 46 km
Correlation
Distance
-1.0
-0.5
0.0
0.5
1.0
0
50
100
150
200
250

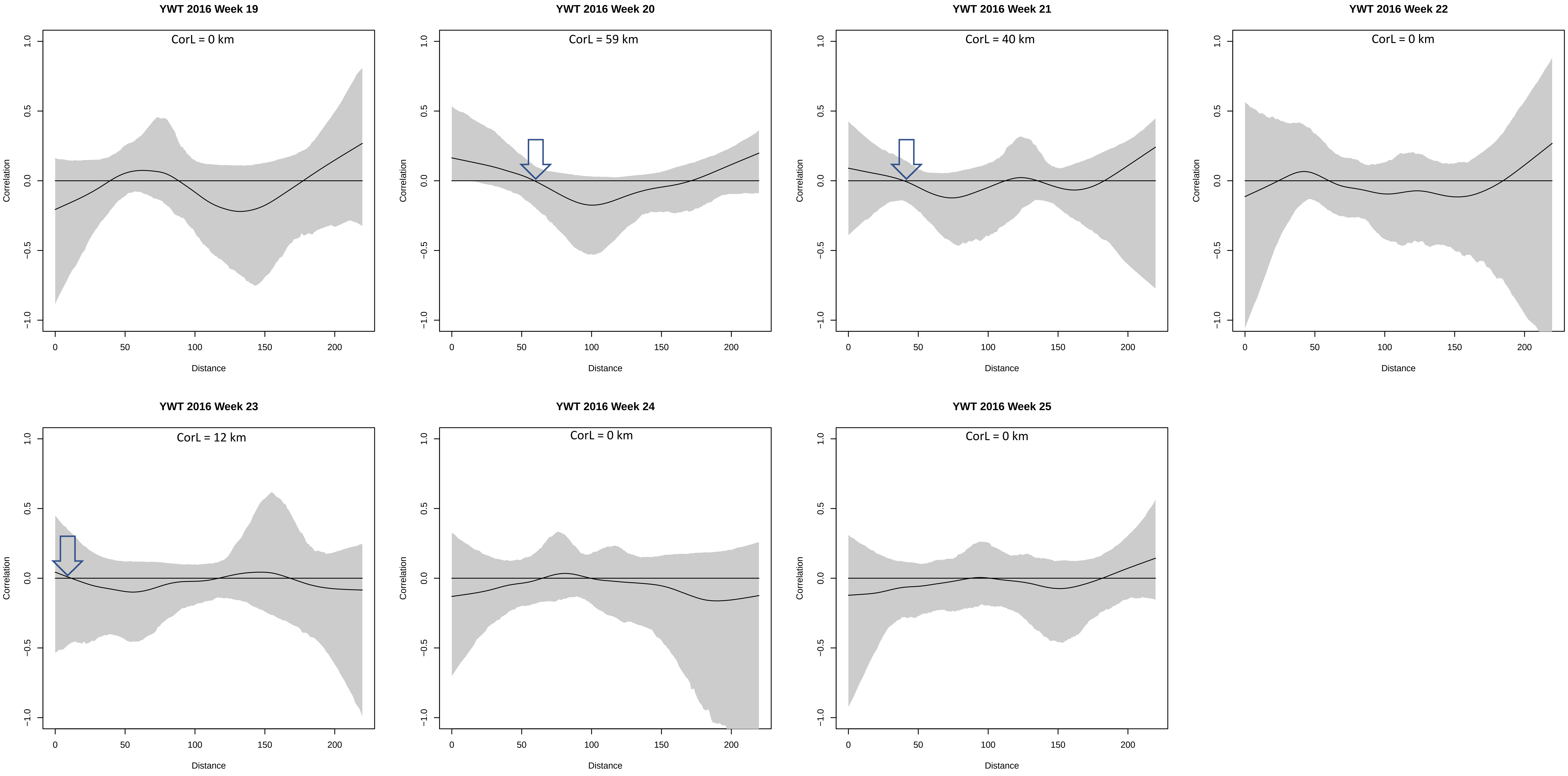
YWT 2016 Week 19
CorL = 0 km
YWT 2016 Week 20
CorL = 59 km
YWT 2016 Week 21
CorL = 40 km
YWT 2016 Week 22
CorL = 0 km
YWT 2016 Week 23
CorL = 12 km
YWT 2016 Week 24
CorL = 0 km
YWT 2016 Week 25
CorL = 0 km
Correlation
Distance

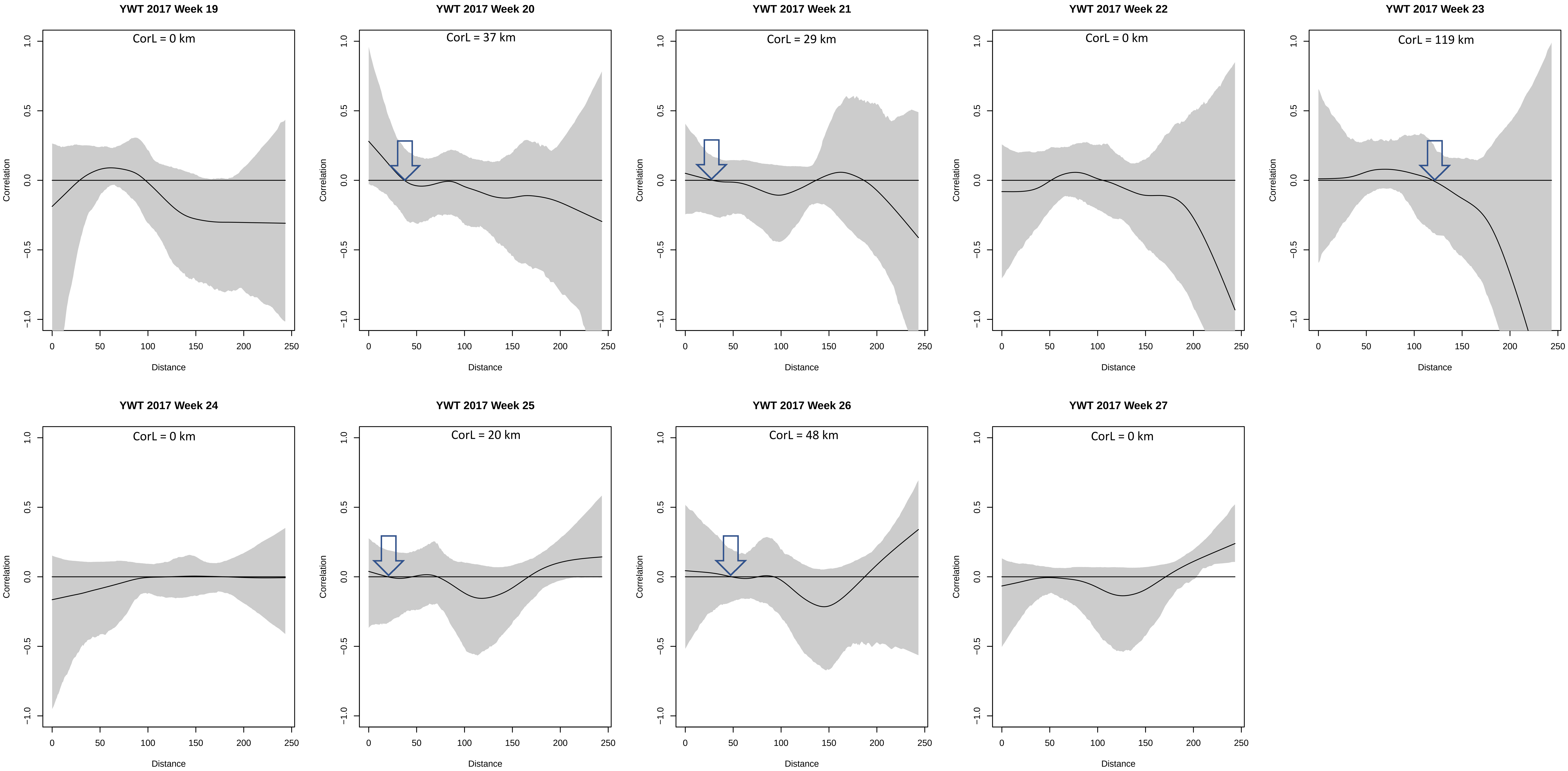

YWT 2017 Week 19
CorL = 0 km
YWT 2017 Week 20
CorL = 37 km
YWT 2017 Week 21
CorL = 29 km
YWT 2017 Week 22
CorL = 0 km
YWT 2017 Week 23
CorL = 119 km
YWT 2017 Week 24
CorL = 0 km
YWT 2017 Week 25
CorL = 20 km
YWT 2017 Week 26
CorL = 48 km
YWT 2017 Week 27
CorL = 0 km
Correlation
Distance

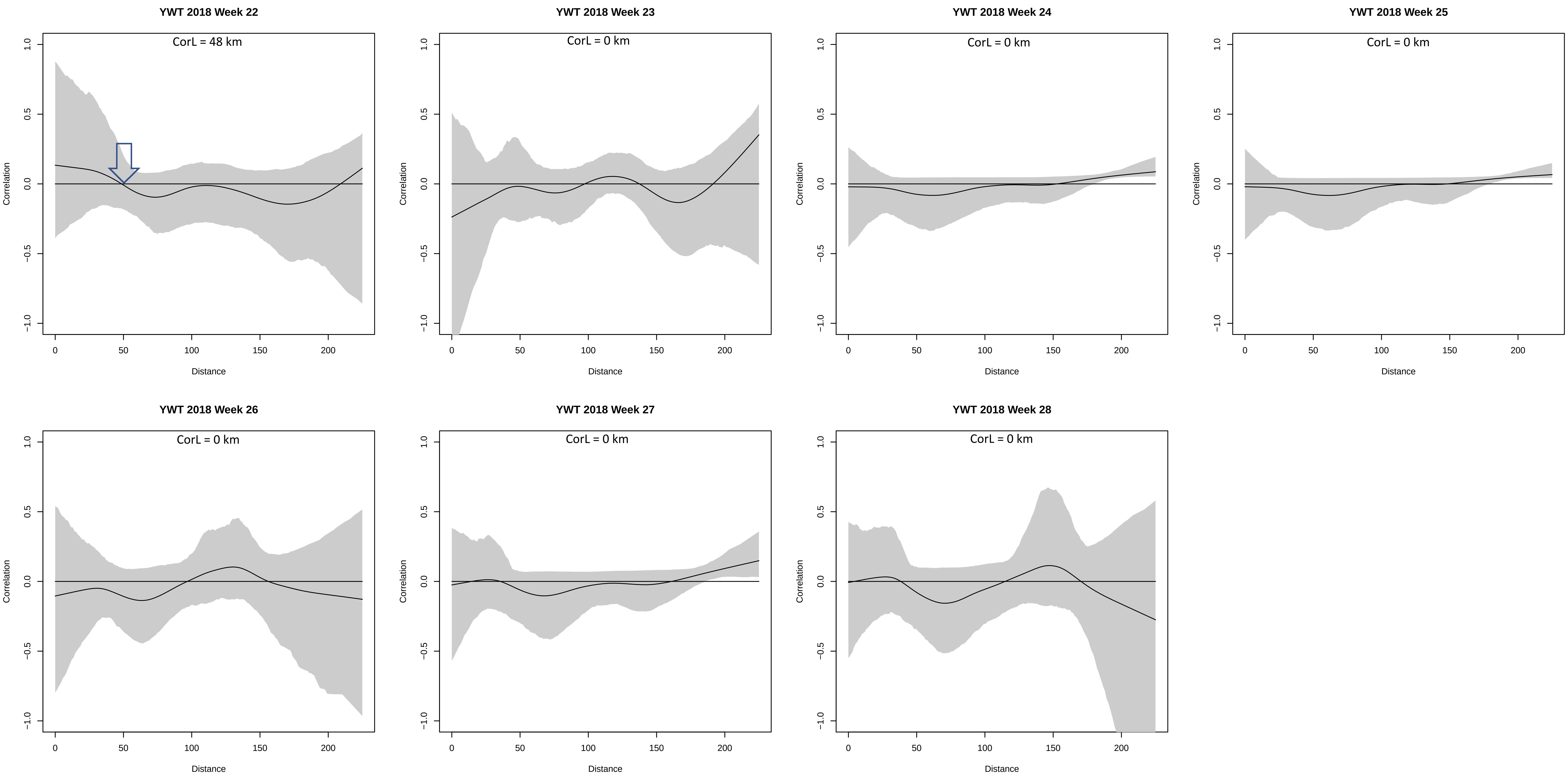
YWT 2018 Week 22
CorL = 48 km
YWT 2018 Week 23
CorL = 0 km
YWT 2018 Week 24
CorL = 0 km
YWT 2018 Week 25
CorL = 0 km
YWT 2018 Week 26
CorL = 0 km
YWT 2018 Week 27
CorL = 0 km
YWT 2018 Week 28
CorL = 0 km
Correlation
Distance
1.0
0.5
0.0
−0.5
−1.0
0
50
100
150
200

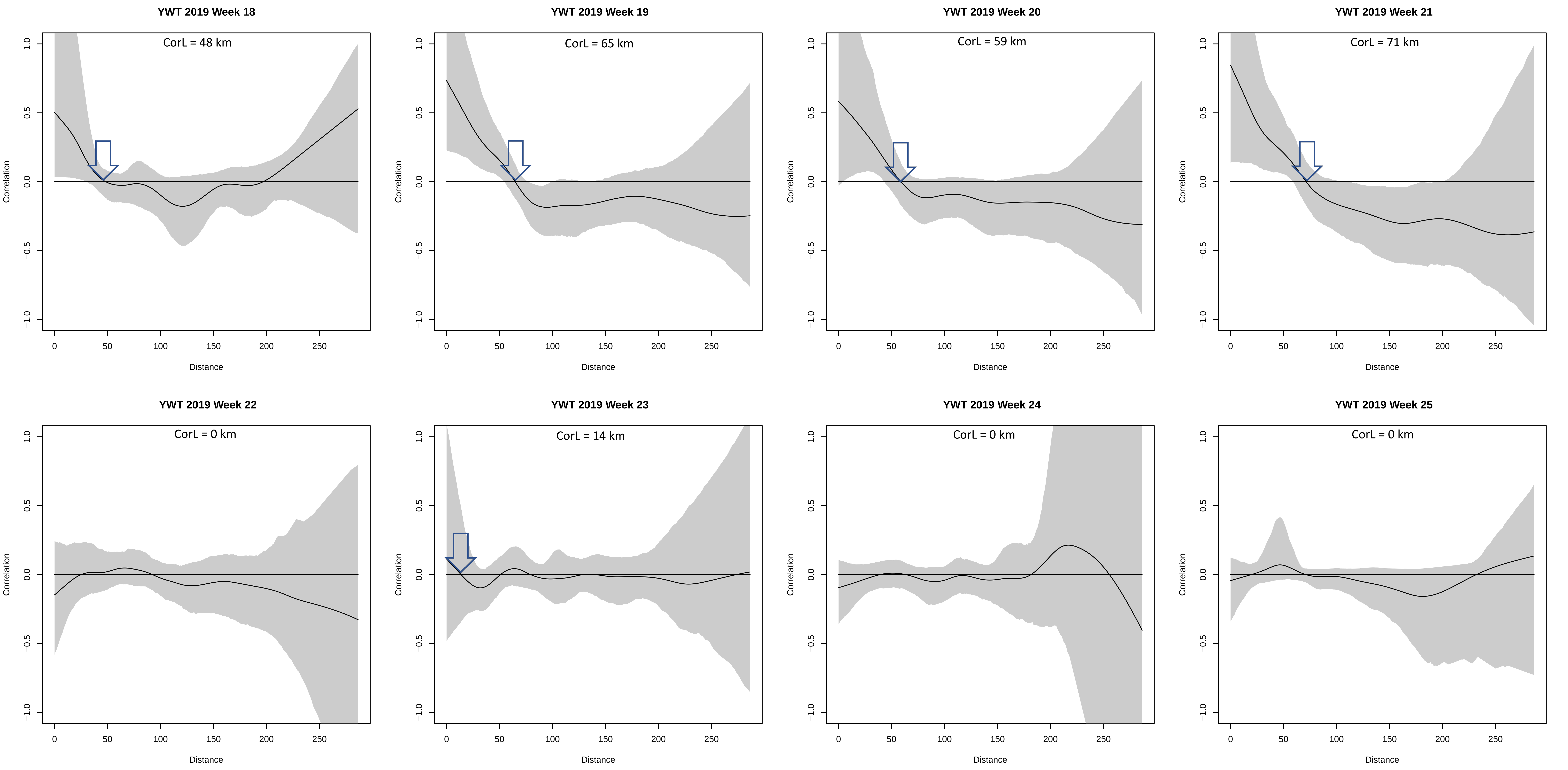
YWT 2019 Week 18
CorL = 48 km
YWT 2019 Week 19
CorL = 65 km
YWT 2019 Week 20
CorL = 59 km
YWT 2019 Week 21
CorL = 71 km
YWT 2019 Week 22
CorL = 0 km
YWT 2019 Week 23
CorL = 14 km
YWT 2019 Week 24
CorL = 0 km
YWT 2019 Week 25
CorL = 0 km
Correlation
Distance

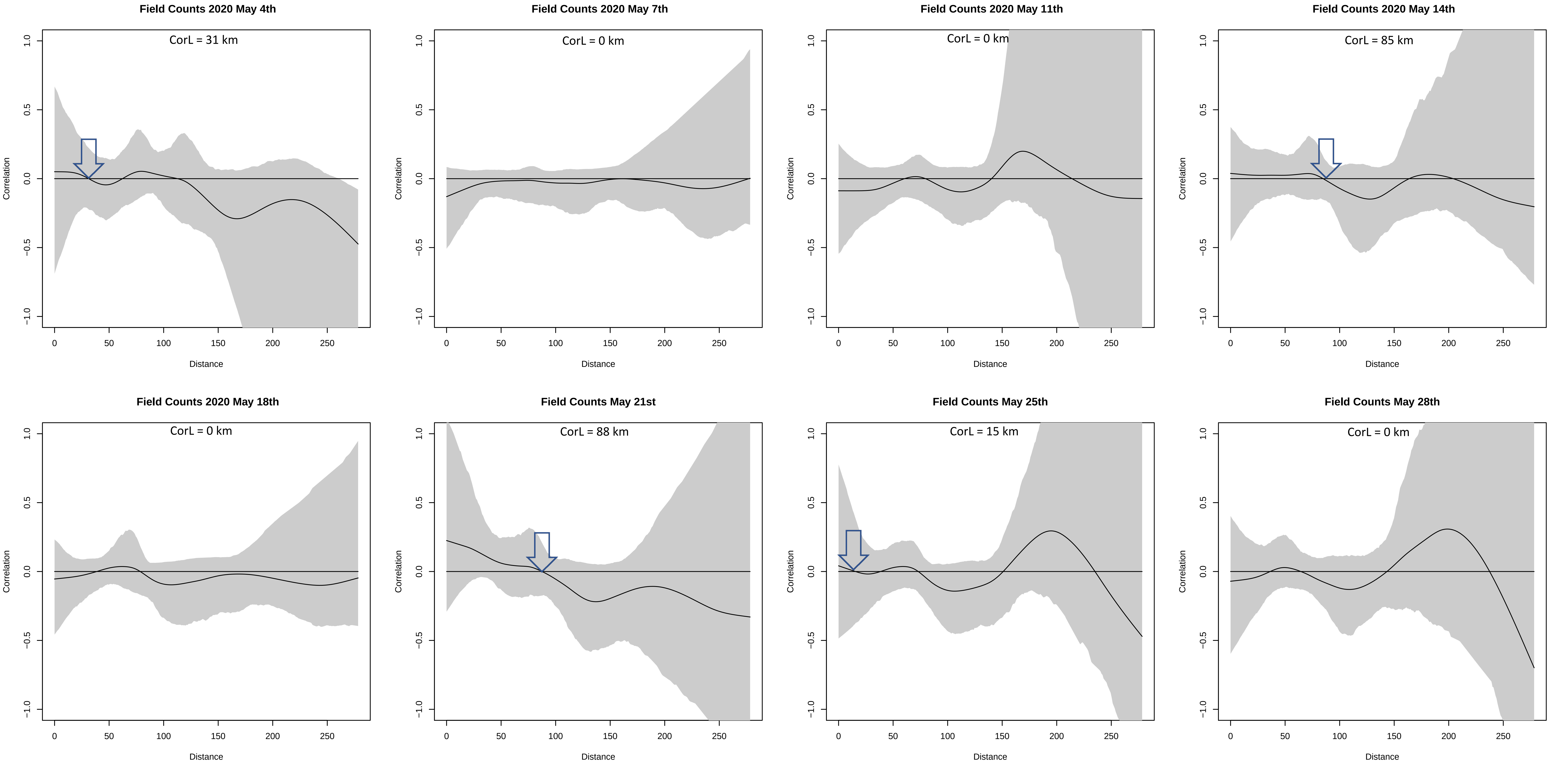
Field Counts 2020 May 4th
CorL = 31 km
Field Counts 2020 May 7th
CorL = 0 km
Field Counts 2020 May 11th
CorL = 0 km
Field Counts 2020 May 14th
CorL = 85 km
Field Counts 2020 May 18th
CorL = 0 km
Field Counts May 21st
CorL = 88 km
Field Counts May 25th
CorL = 15 km
Field Counts May 28th
CorL = 0 km
Correlation
Distance

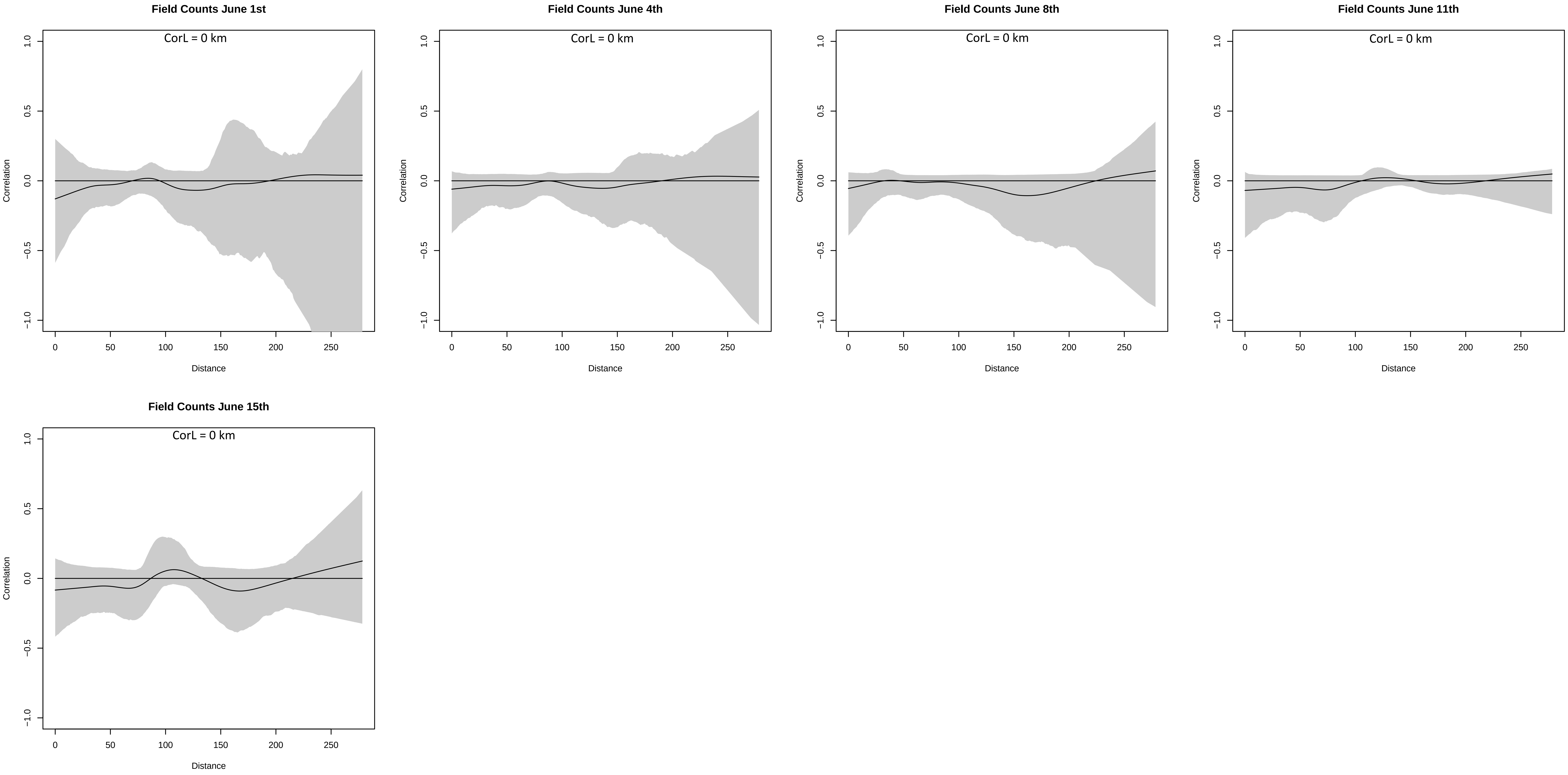
Field Counts June 1st
CorL = 0 km
Field Counts June 4th
CorL = 0 km
Field Counts June 8th
CorL = 0 km
Field Counts June 11th
CorL = 0 km
Field Counts June 15th
CorL = 0 km
Correlation
Distance
−1.0
−0.5
0.0
0.5
1.0
0
50
100
150
200
250
