# Supplementary material for: Quantifying inherent predictability and spatial synchrony in the aphid vector Myzus persicae: field‐scale patterns of abundance and regional forecasting error in the UK
Source: Pest Manag Sci. 2022 Dec 19;79(4):1331–41. doi: 10.1002/ps.7292 (PMC10952309; doi:10.1002/ps.7292)

Spatial Generalized Additive Mixed Models
YWT 2014-2019 and Crop Inspection Data 2020

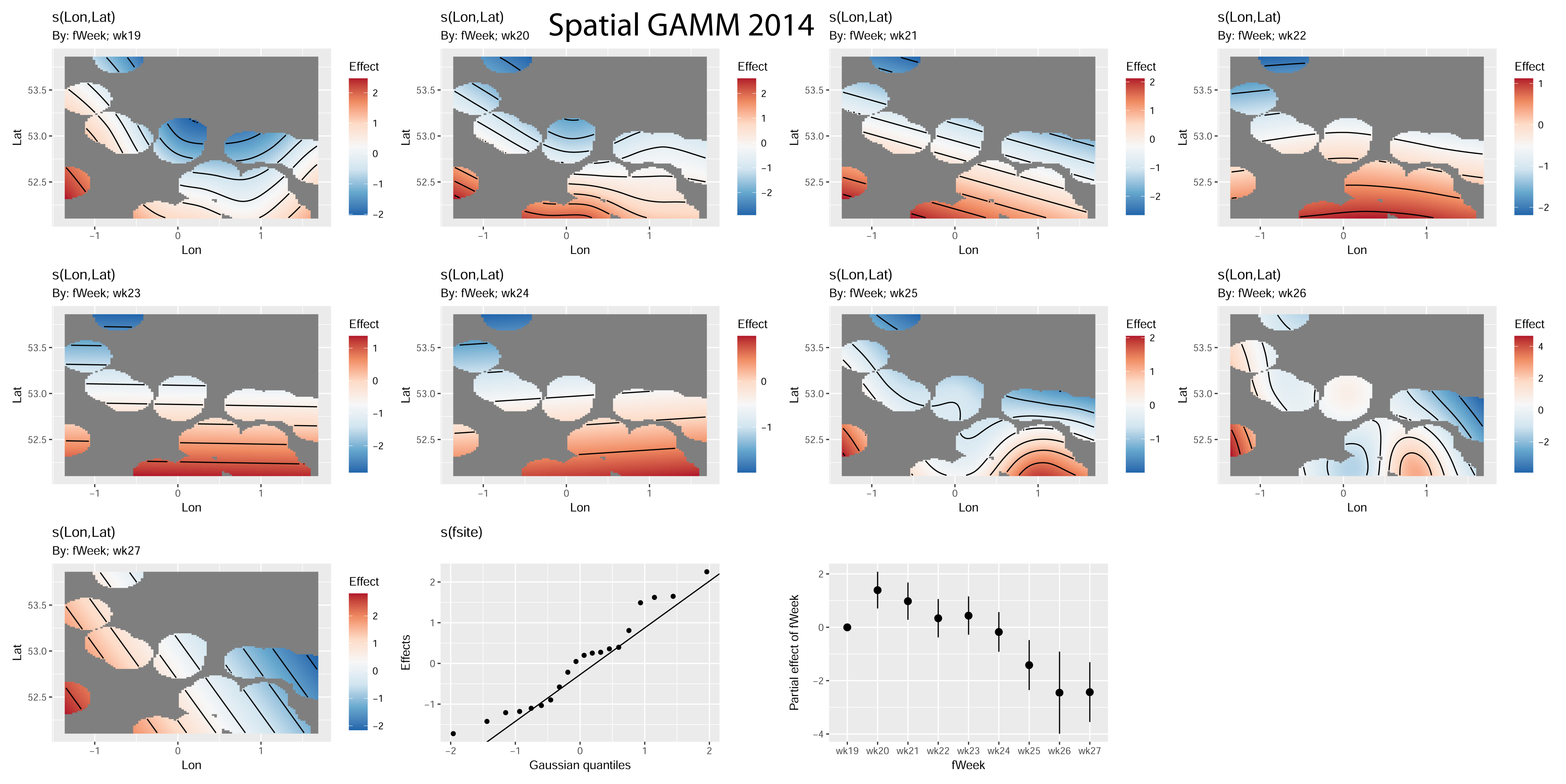
Spatial GAMM 2014
s(Lon,Lat)
By: fWeek; wk19
s(Lon,Lat)
By: fWeek; wk20
s(Lon,Lat)
By: fWeek; wk21
s(Lon,Lat)
By: fWeek; wk22
s(Lon,Lat)
By: fWeek; wk23
s(Lon,Lat)
By: fWeek; wk24
s(Lon,Lat)
By: fWeek; wk25
s(Lon,Lat)
By: fWeek; wk26
s(Lon,Lat)
By: fWeek; wk27
Effect
Lat
Lon
s(fsite)
Effects
Gaussian quantiles
Partial effect of fWeek
fWeek
wk19
wk20
wk21
wk22
wk23
wk24
wk25
wk26
wk27

Spatial GAMM 2014

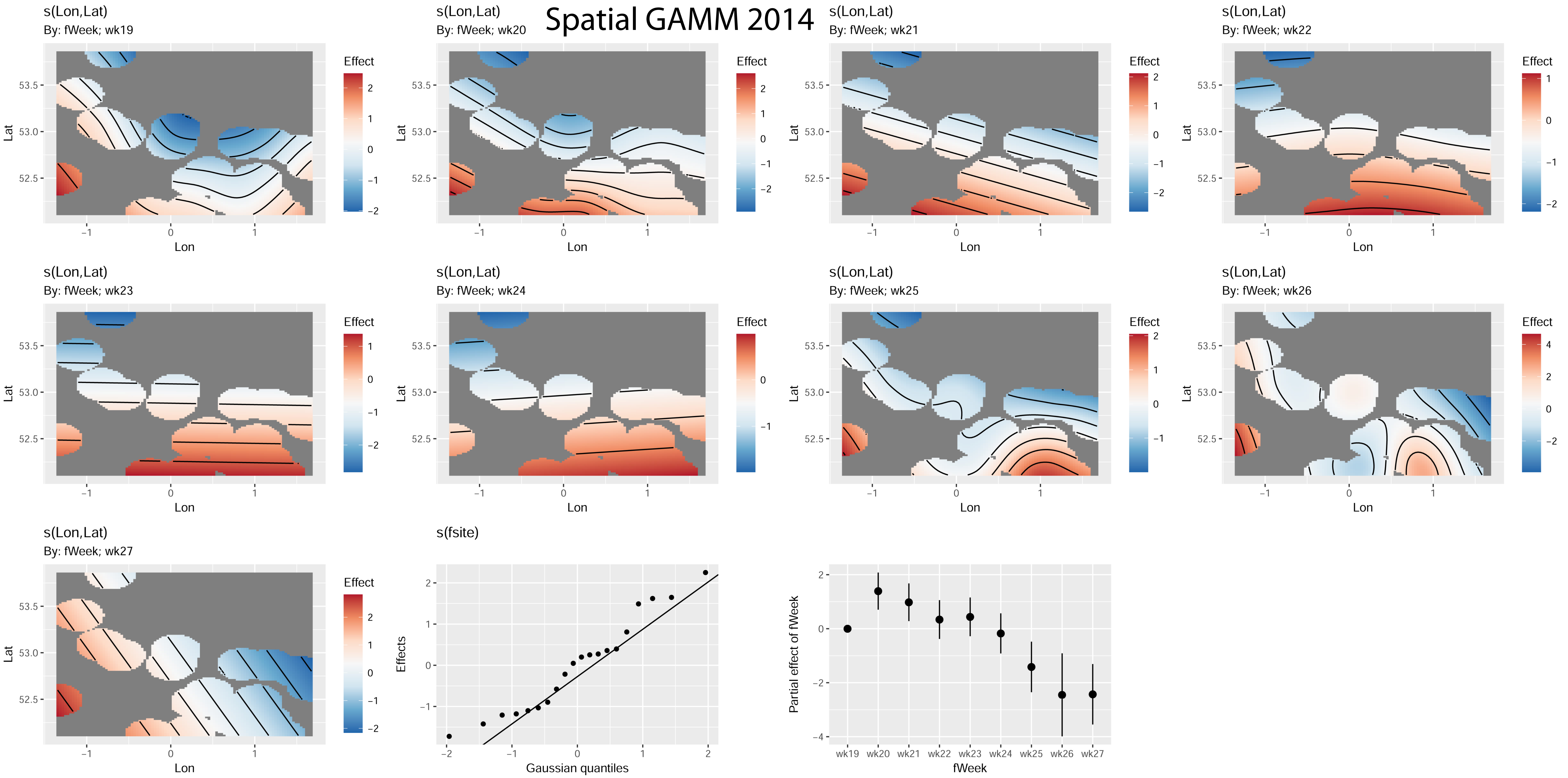

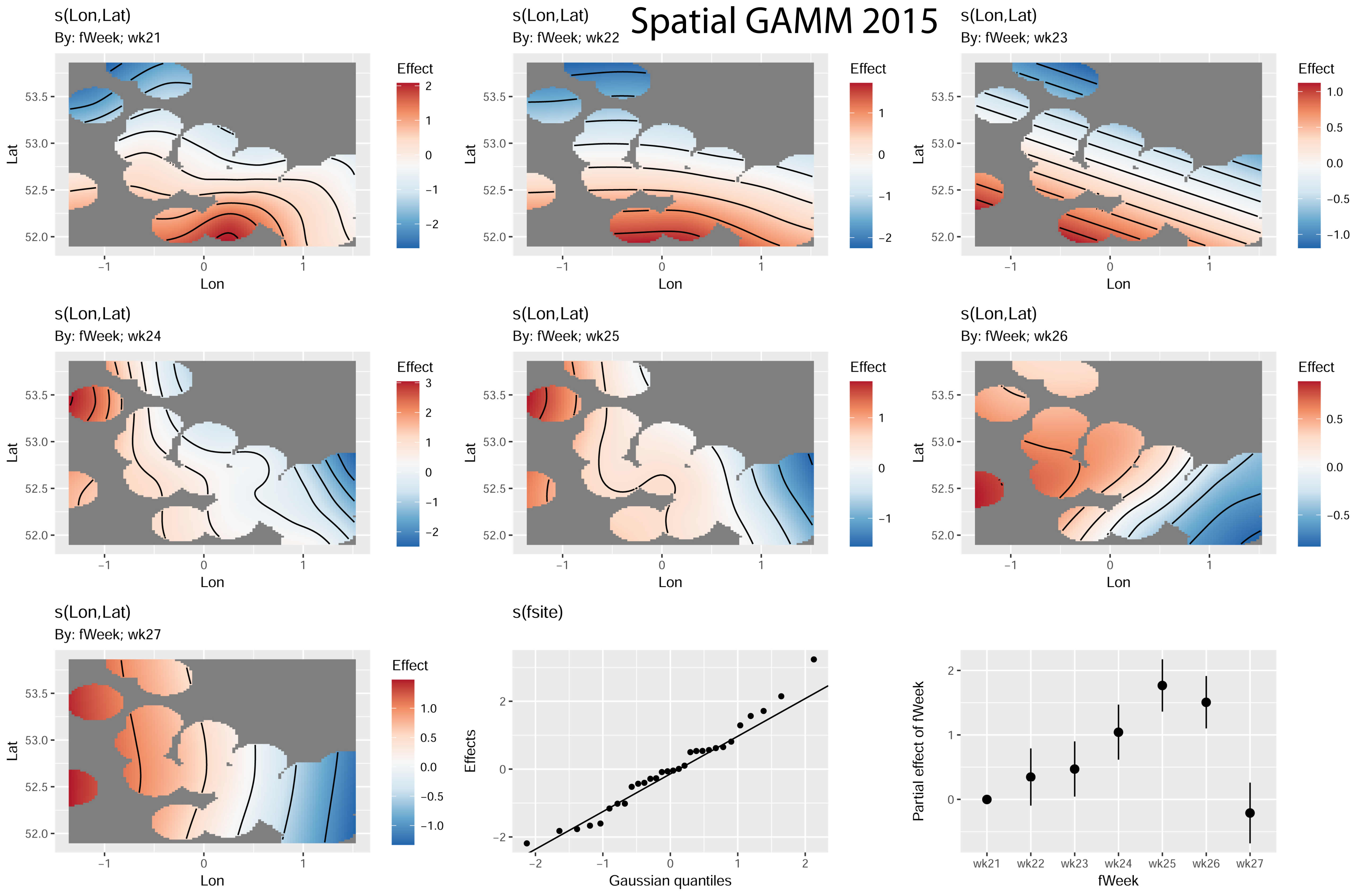

Spatial GAMM 2015
s(Lon,Lat)
By: fWeek; wk21
s(Lon,Lat)
By: fWeek; wk22
s(Lon,Lat)
By: fWeek; wk23
s(Lon,Lat)
By: fWeek; wk24
s(Lon,Lat)
By: fWeek; wk25
s(Lon,Lat)
By: fWeek; wk26
s(Lon,Lat)
By: fWeek; wk27
Effect
Lat
Lon
s(fsite)
Effects
Gaussian quantiles
Partial effect of fWeek
fWeek
wk21
wk22
wk23
wk24
wk25
wk26
wk27

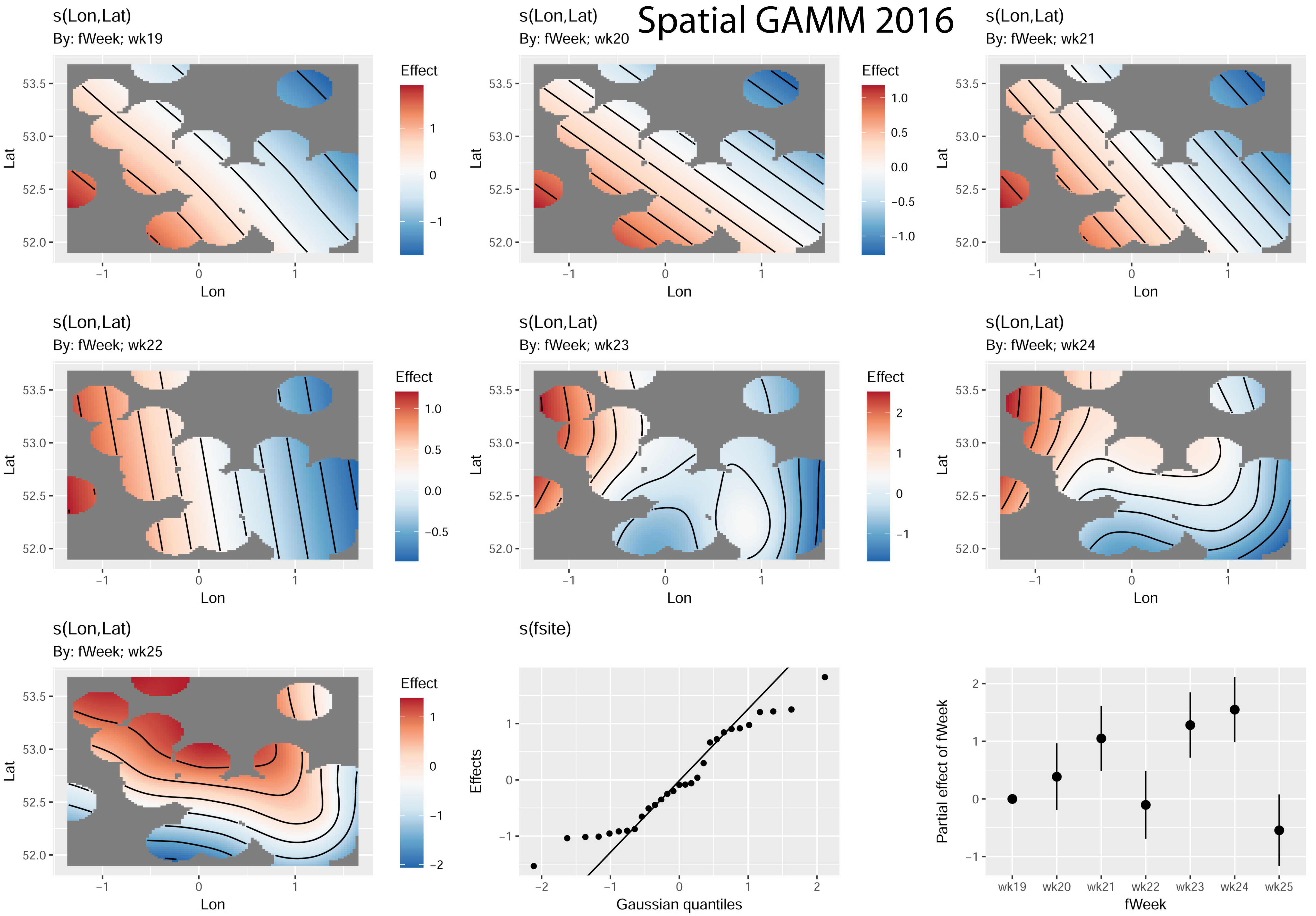
Spatial GAMM 2016
s(Lon,Lat)
By: fWeek; wk19
s(Lon,Lat)
By: fWeek; wk20
s(Lon,Lat)
By: fWeek; wk21
s(Lon,Lat)
By: fWeek; wk22
s(Lon,Lat)
By: fWeek; wk23
s(Lon,Lat)
By: fWeek; wk24
s(Lon,Lat)
By: fWeek; wk25
Effect
Lat
Lon
53.5
53.0
52.5
52.0
−1
0
1
s(fsite)
Effects
Gaussian quantiles
Partial effect of fWeek
fWeek
wk19
wk20
wk21
wk22
wk23
wk24
wk25

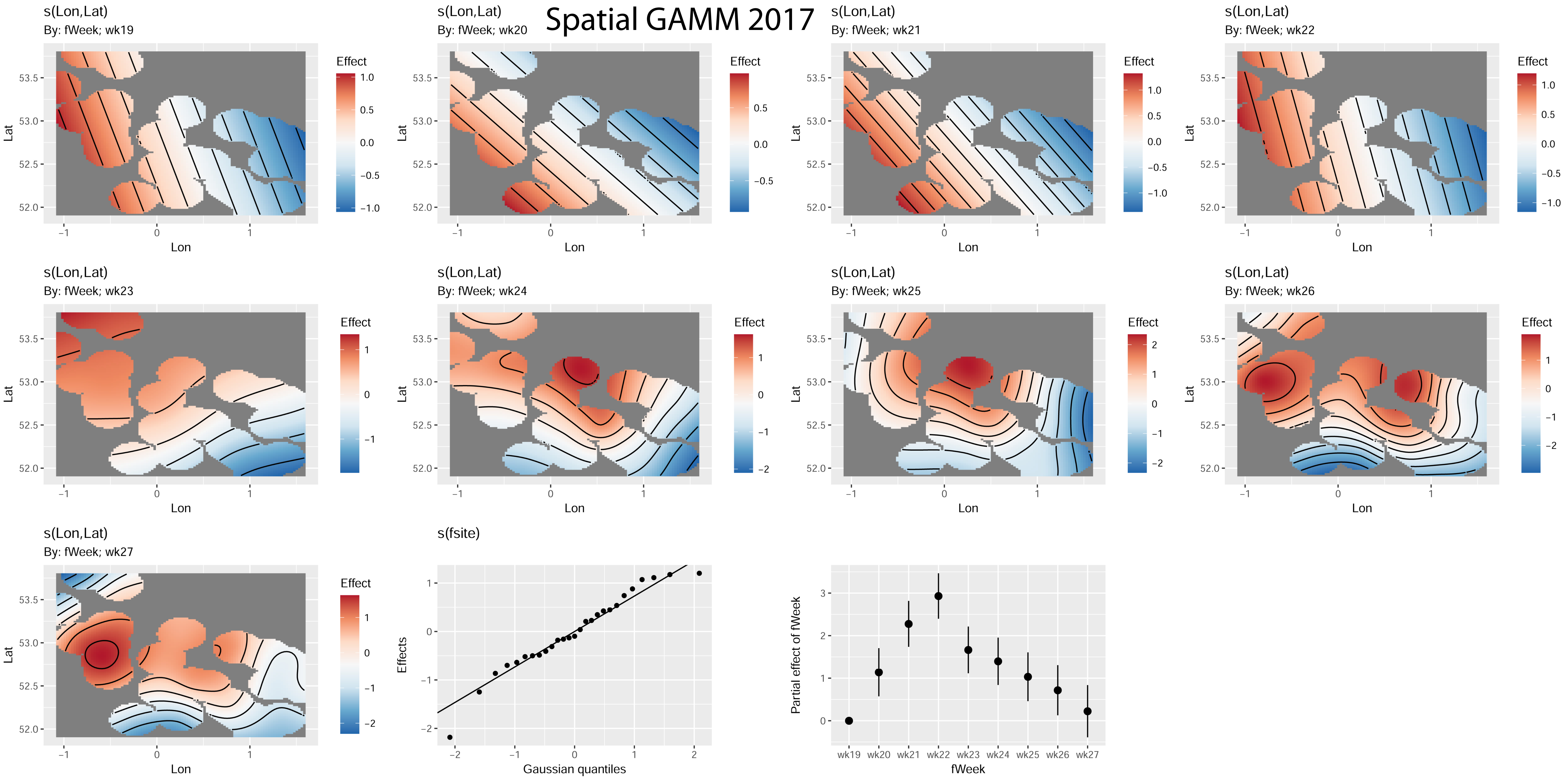
Spatial GAMM 2017
s(Lon,Lat)
By: fWeek; wk19
s(Lon,Lat)
By: fWeek; wk20
s(Lon,Lat)
By: fWeek; wk21
s(Lon,Lat)
By: fWeek; wk22
s(Lon,Lat)
By: fWeek; wk23
s(Lon,Lat)
By: fWeek; wk24
s(Lon,Lat)
By: fWeek; wk25
s(Lon,Lat)
By: fWeek; wk26
s(Lon,Lat)
By: fWeek; wk27
Effect
Lat
Lon
s(fsite)
Effects
Gaussian quantiles
Partial effect of fWeek
fWeek
wk19
wk20
wk21
wk22
wk23
wk24
wk25
wk26
wk27

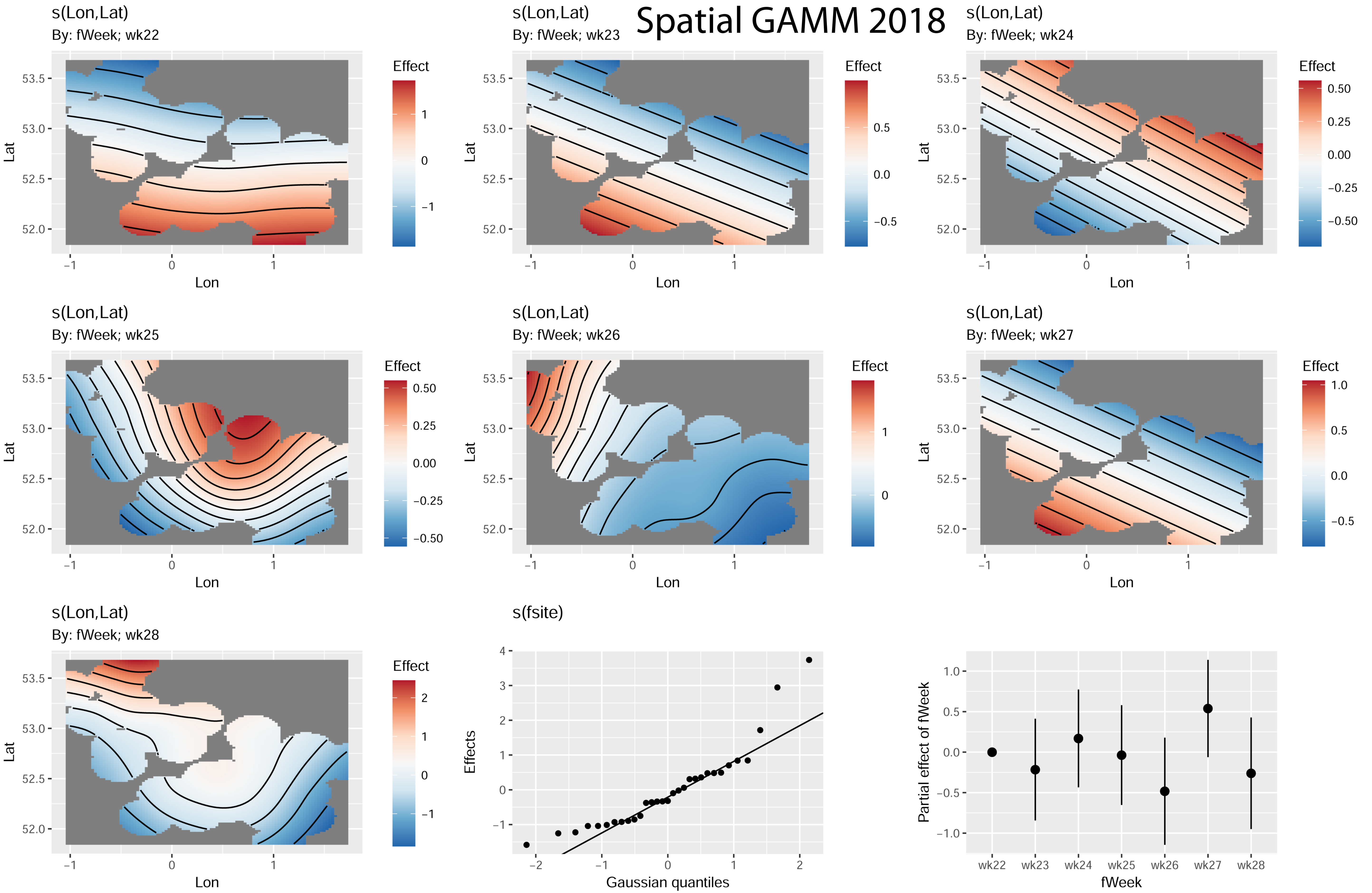
Spatial GAMM 2018
s(Lon,Lat)
By: fWeek; wk22
s(Lon,Lat)
By: fWeek; wk23
s(Lon,Lat)
By: fWeek; wk24
s(Lon,Lat)
By: fWeek; wk25
s(Lon,Lat)
By: fWeek; wk26
s(Lon,Lat)
By: fWeek; wk27
s(Lon,Lat)
By: fWeek; wk28
Effect
Lat
Lon
s(fsite)
Effects
Gaussian quantiles
Partial effect of fWeek
fWeek
wk22
wk23
wk24
wk25
wk26
wk27
wk28

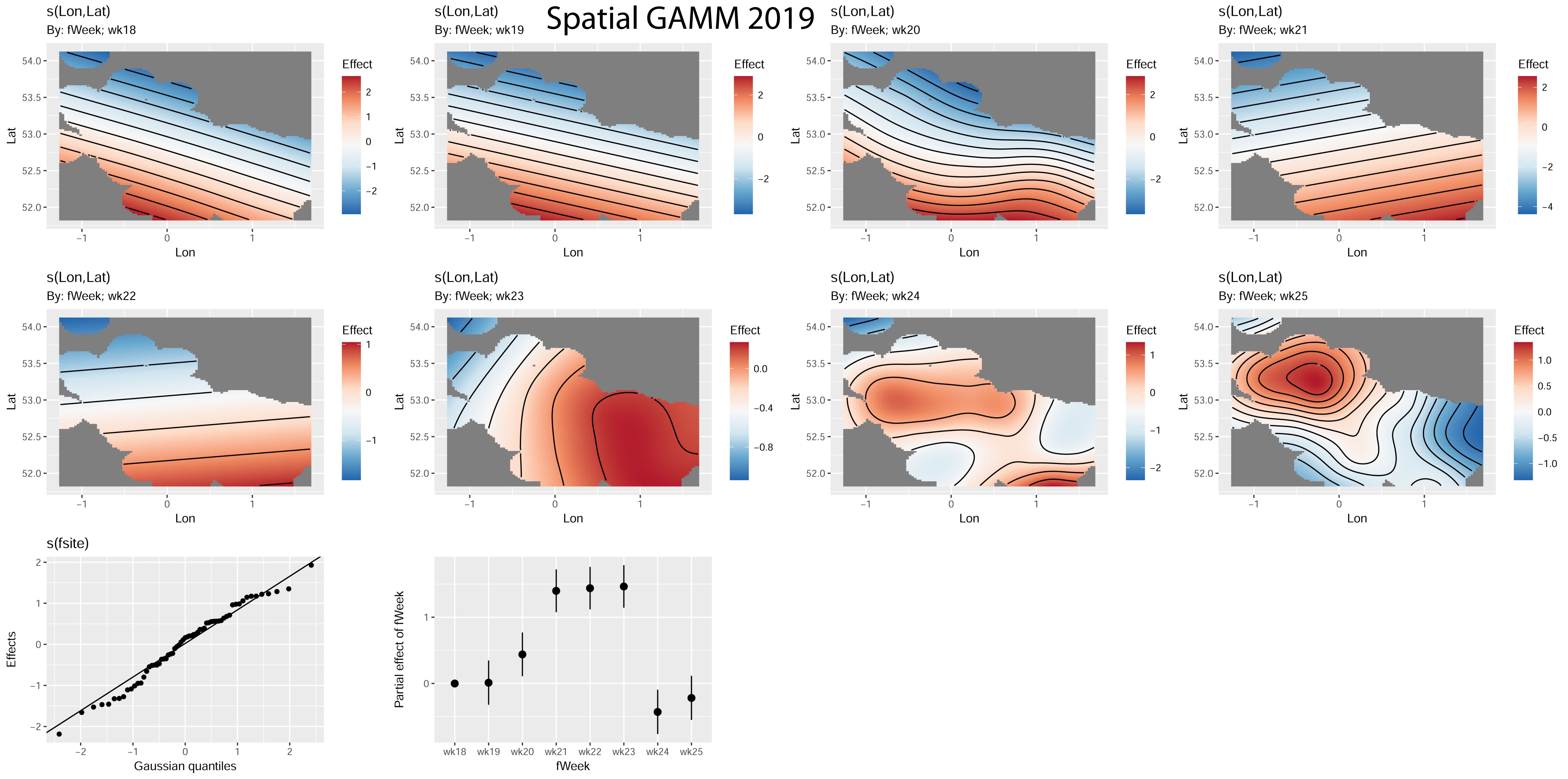
Spatial GAMM 2019
s(Lon,Lat)
By: fWeek; wk18
s(Lon,Lat)
By: fWeek; wk19
s(Lon,Lat)
By: fWeek; wk20
s(Lon,Lat)
By: fWeek; wk21
s(Lon,Lat)
By: fWeek; wk22
s(Lon,Lat)
By: fWeek; wk23
s(Lon,Lat)
By: fWeek; wk24
s(Lon,Lat)
By: fWeek; wk25
Effect
Lat
Lon
s(fsite)
Effects
Gaussian quantiles
Partial effect of fWeek
fWeek
wk18
wk19
wk20
wk21
wk22
wk23
wk24
wk25

Spatial GAMM 2020

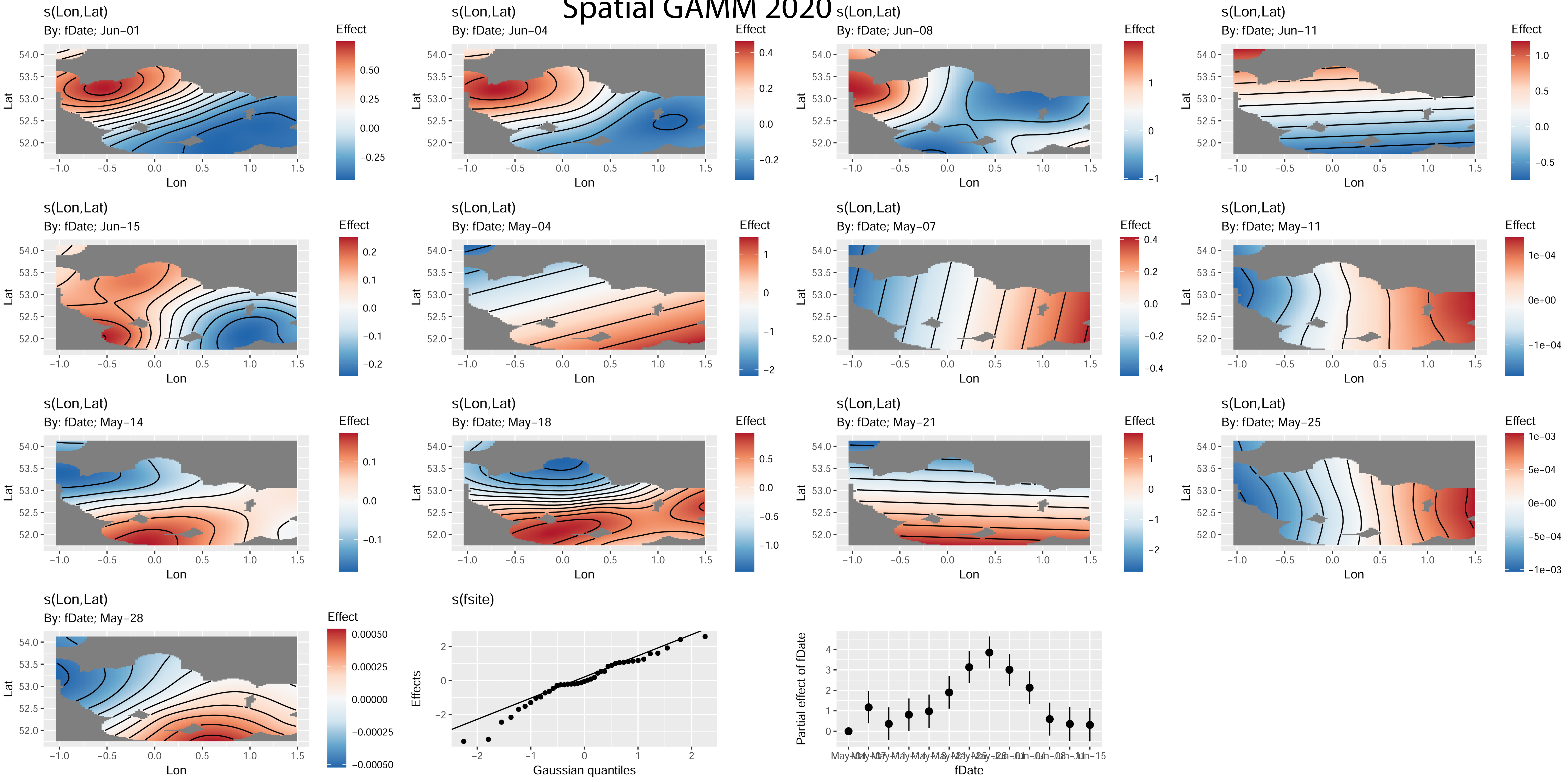

Supplement: Supplementary file 4 — Figures S4: Spatial Generalized Additive Mixed Models [file PS-79-1331-s006.pdf]
